# Supplementary material for: Genome-wide QTL mapping of yield and agronomic traits in two widely adapted winter wheat cultivars from multiple mega-environments
Source: PeerJ. 2021 Nov 24;9:e12350. doi: 10.7717/peerj.12350 (PMC8627123; doi:10.7717/peerj.12350)
Supplement: Supplemental Information 2 [file peerj-09-12350-s002.docx]

Supplemental Fig. S1 Boxplot analysis of yield and agronomic traits. Traits included are a) Heading date, b) Plant height, c) Test weight, d) Grain yield. X-axis is individual environment: Texas A&M AgriLife Research station in Bushland, TX in 2011, 2012, 2017 as dryland (11BD, 12BD, 17BD), irrigated in 2017 (17BI), Chillicothe, TX in 2011, 2012 and 2014 as dryland (11CH, 12CH, 14CH), Etter, TX with five irrigation levels (40%, 50%, 65%,75% and 100%) in 2011 (11EP1, 11EP2, 11EP3, 11EP4, 11EP5), three irrigation levels in 2012 (12EP1, 12EP2, 12EP3), four irrigation levels in 2013 (13EP2, 13EP3, 13EP4, 13EP5), two irrigation levels in 2014 (14EP4, 14EP5), and irrigated in 2017 (17EI), Uvalde, TX in 2012 as dryland (12UVLD = 12UVD), and with three irrigation levels in 2012 (50%, 75%, 100%) (12UV5, 12UV7, 12UVL), irrigated in 2013 (13UVL), Clovis, NM irrigated in 2017 (17CVI); Y-axis represents the corresponding trait value in the respective environment. Descriptive statistics are on top of each boxplot.


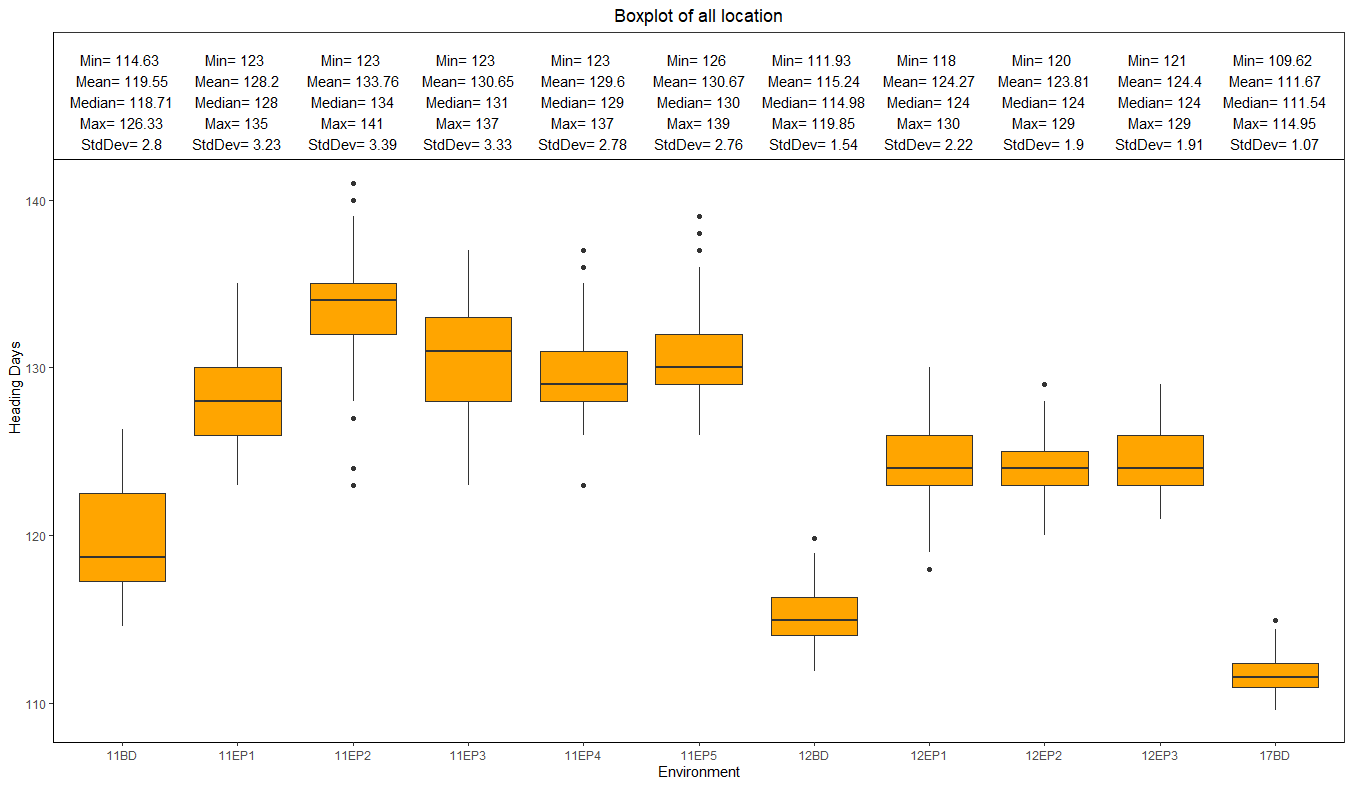


**a)**


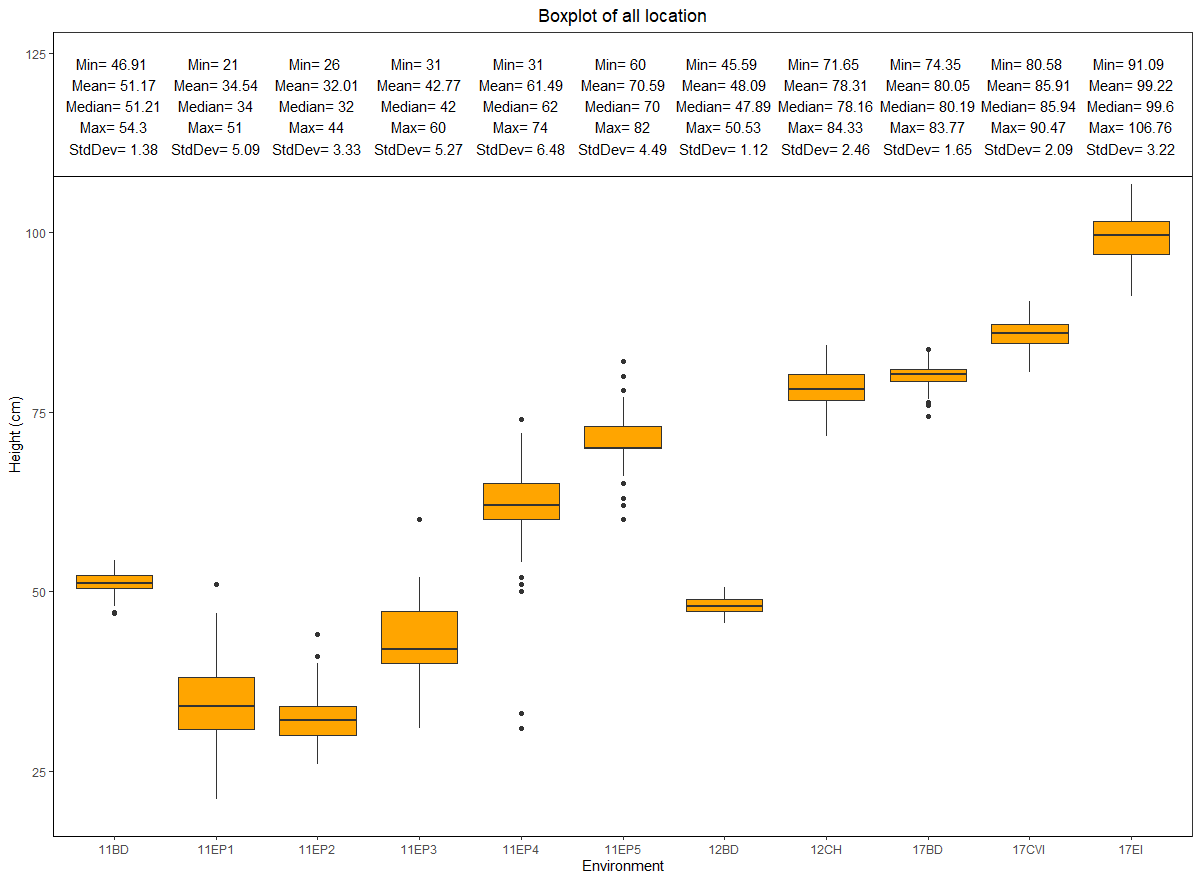


**b)**

**c)**


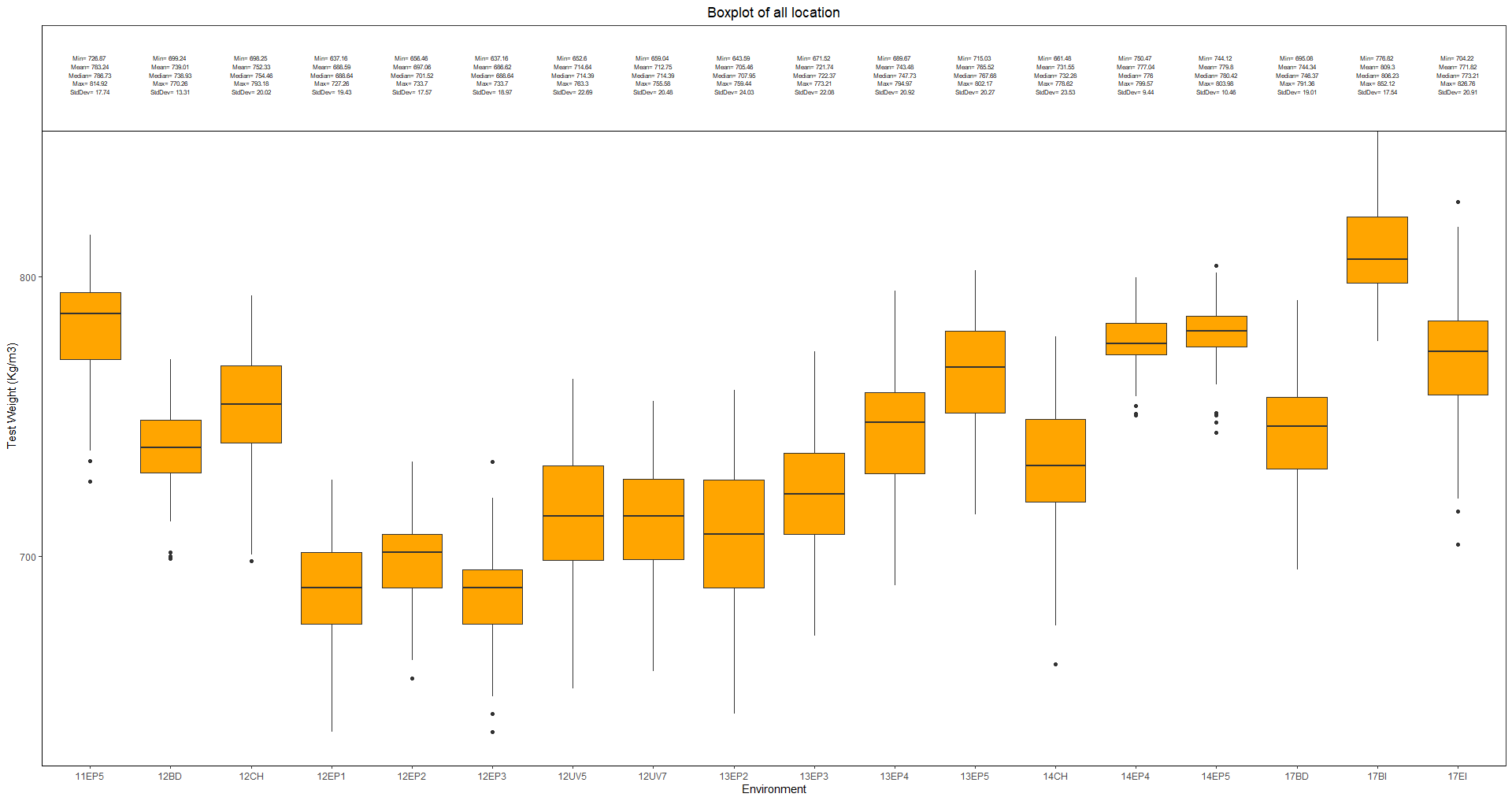


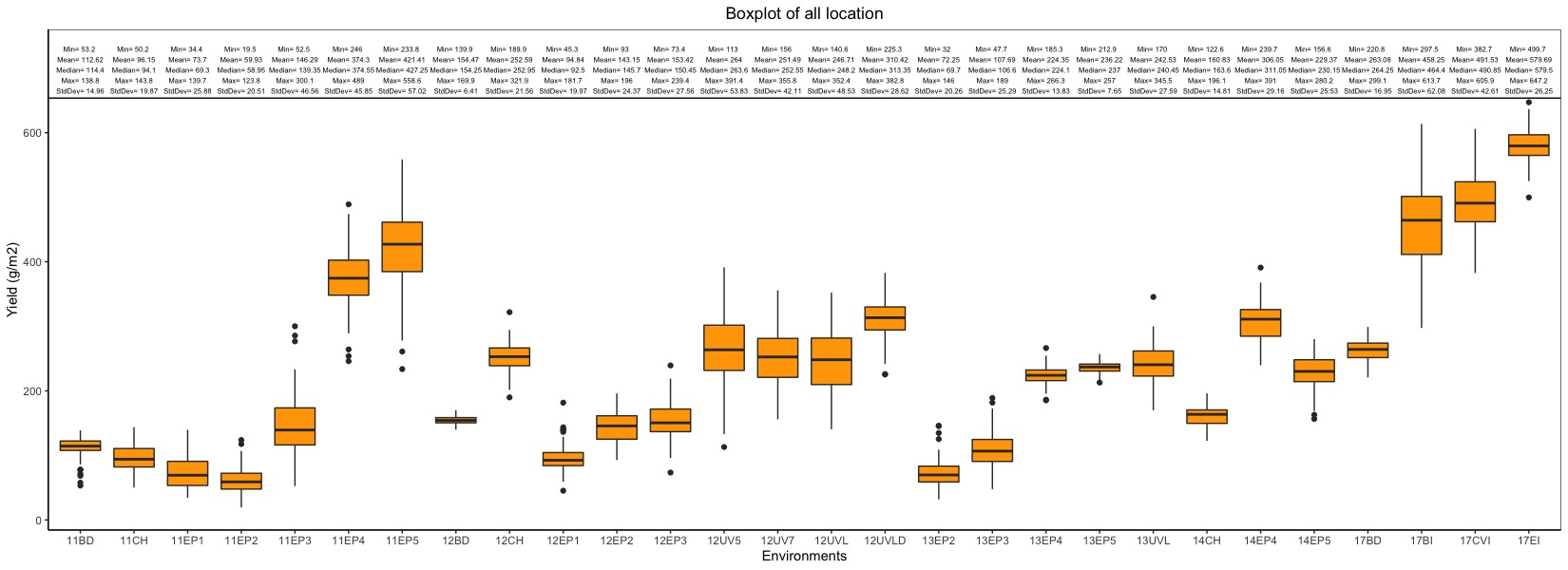


**d)**

Supplemental Fig. S2 LOD profile and additive effects of QTLs detected in the environment 1) 11BD, 2) 11EP1, 3) 11EP2, 4) 11EP3, 5) 11EP4, 6) 11EP5, 7) 12BD, 8) 12CH, 9) 12EP1, 10) 12EP2, 11) 12EP3, 12) 12UV5, 13) 12UV7, 14) 12UVL, 15) 12UVLD, 16) 13EP2, 17) 13EP3, 18) 13EP4, 19) 13EP5, 20) 13UVL, 21) 14CH, 22) 14EP4, 23) 14EP5, 24) 17BD, 25) 7BI, 26) 17CVI, 27) 17EI. Env 11CH was excluded due to only one trait. Top figure shows LOD profile with chromosomal position (cM of 25 linkage groups across 21 chromosomes) along the x-axis and LOD score on the y-axis. Bottom figures show additive effect profile with chromosomal position along the x-axis and additive effects on the y-axis for traits with significant QTL. Different traits are color coded as shown on the top of each panel.


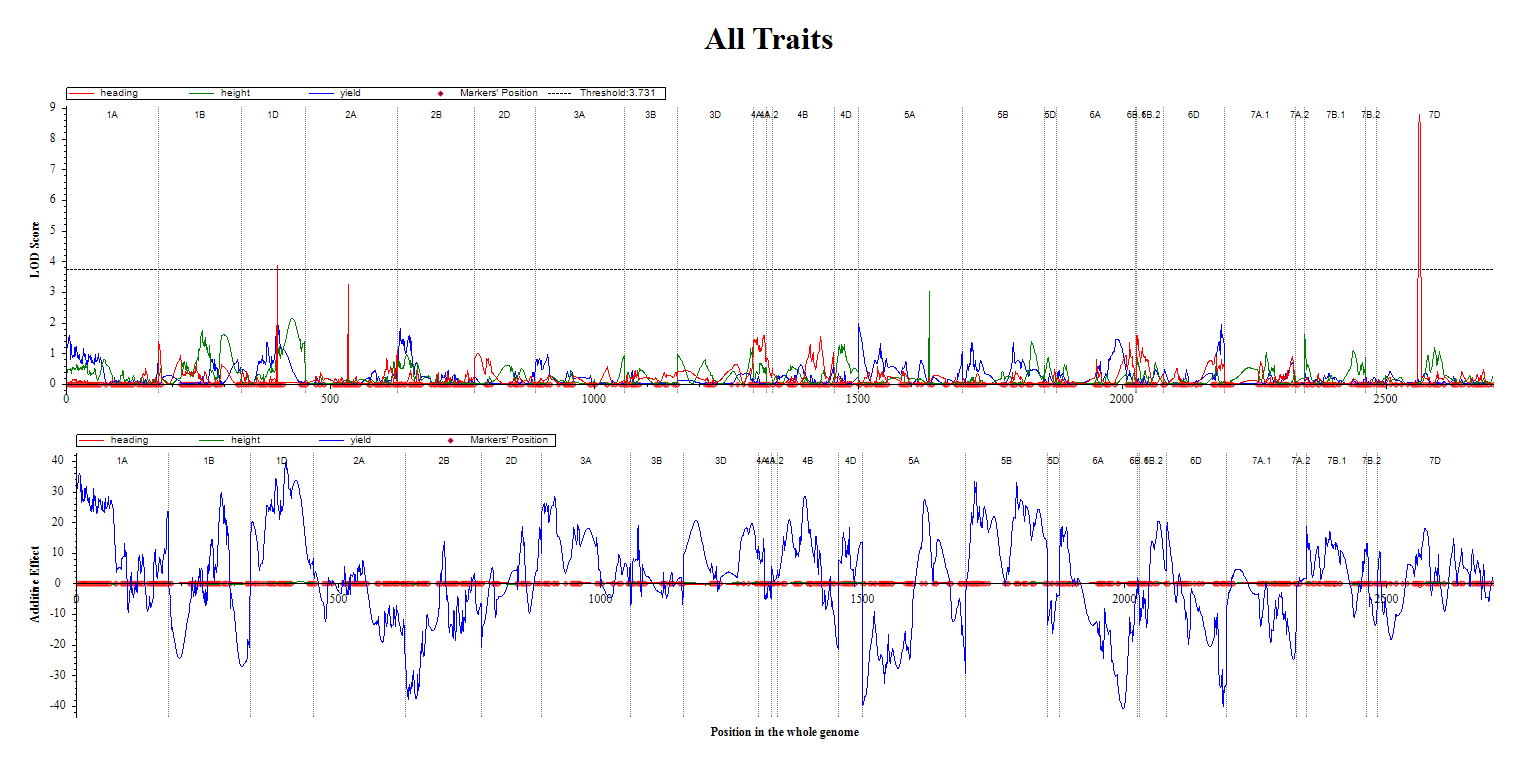


**1)**


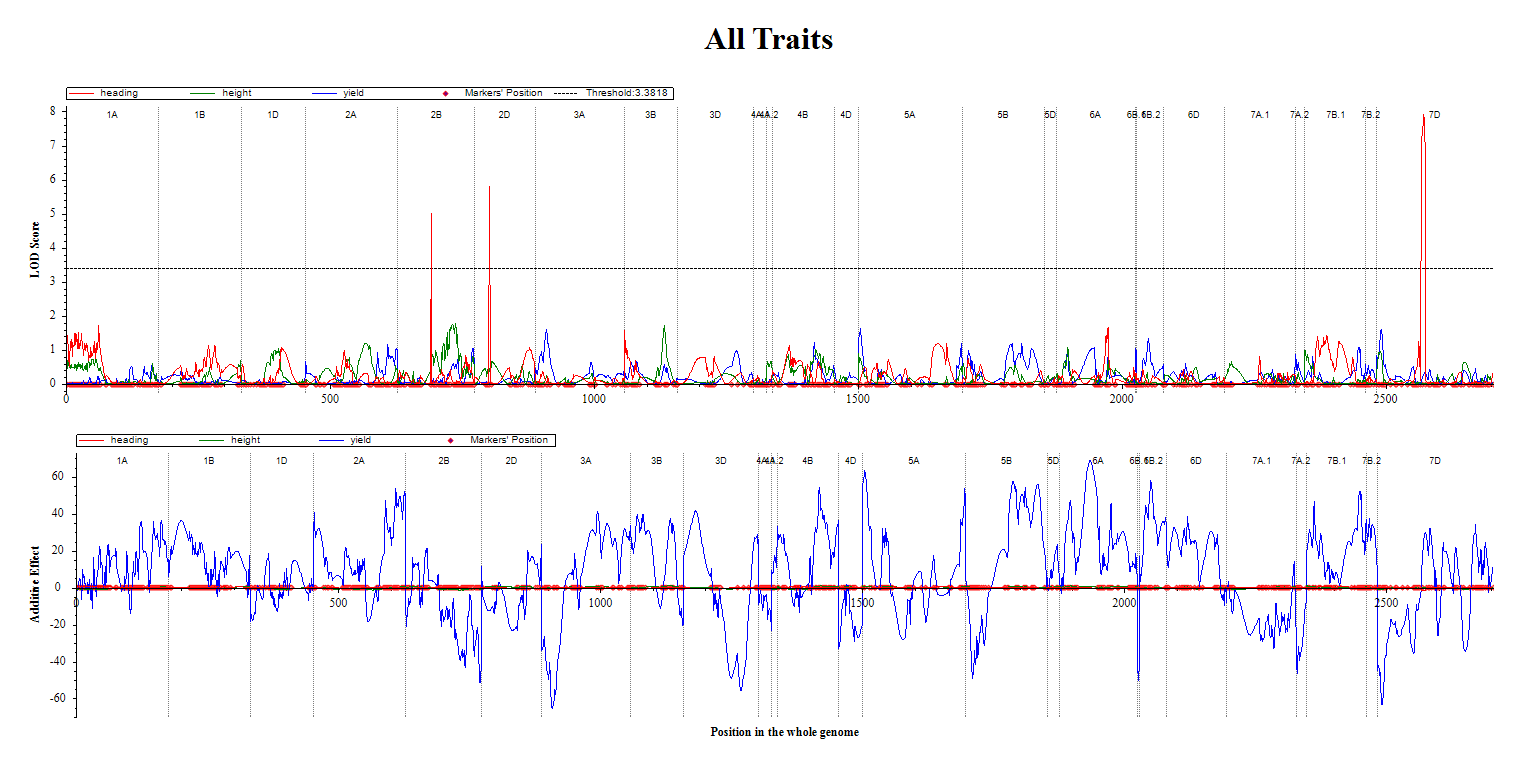


**2)**


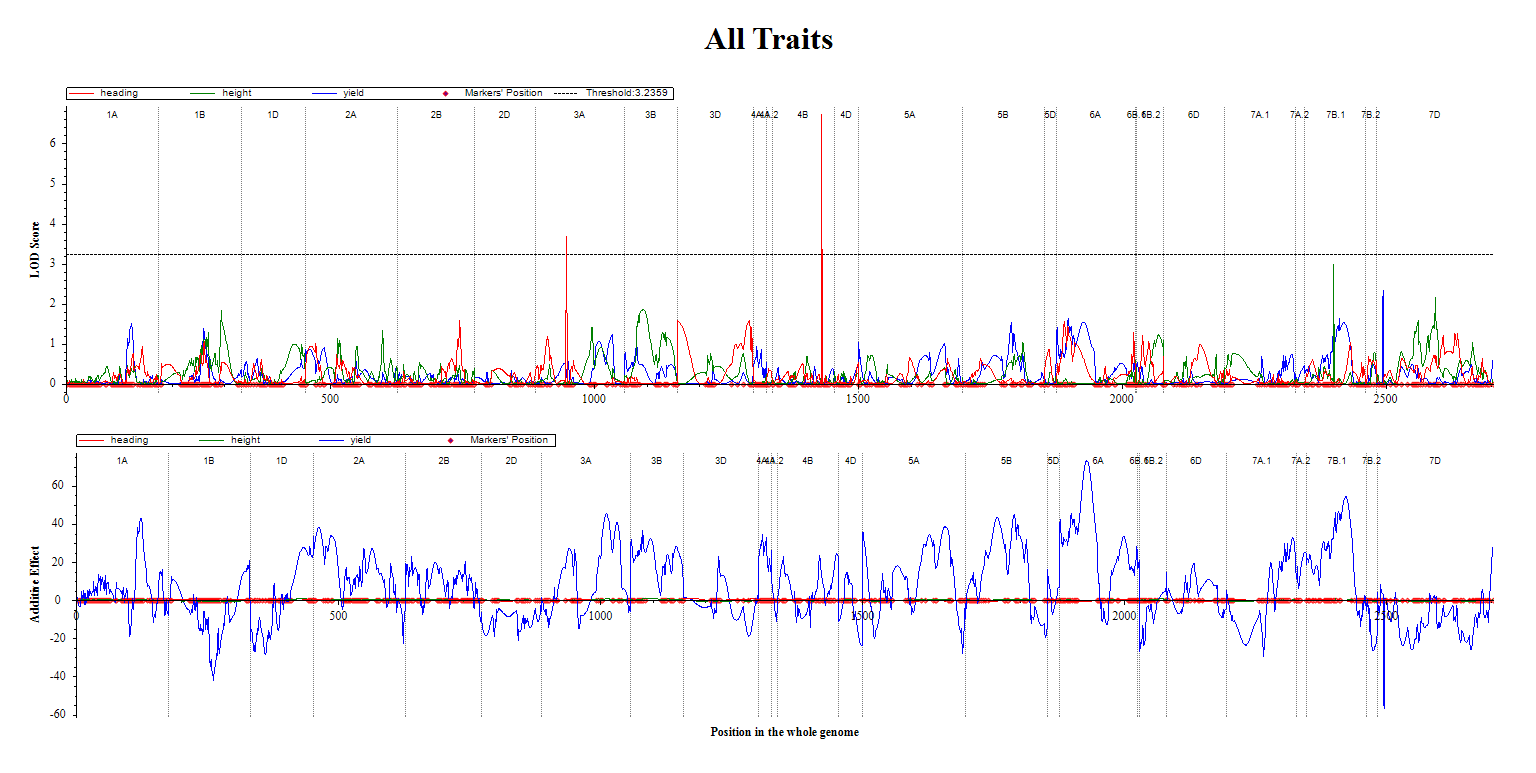


**3)**


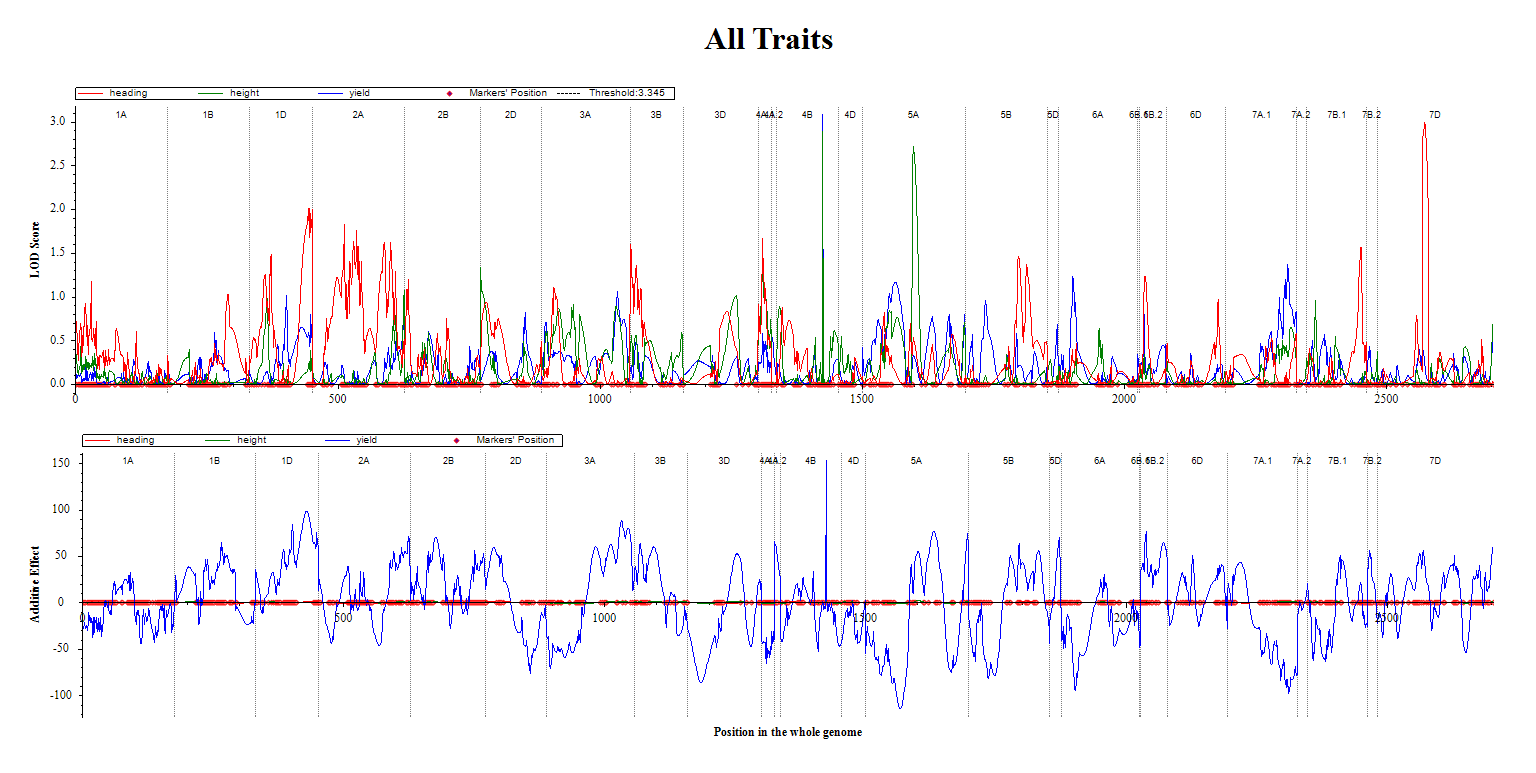


**4)**


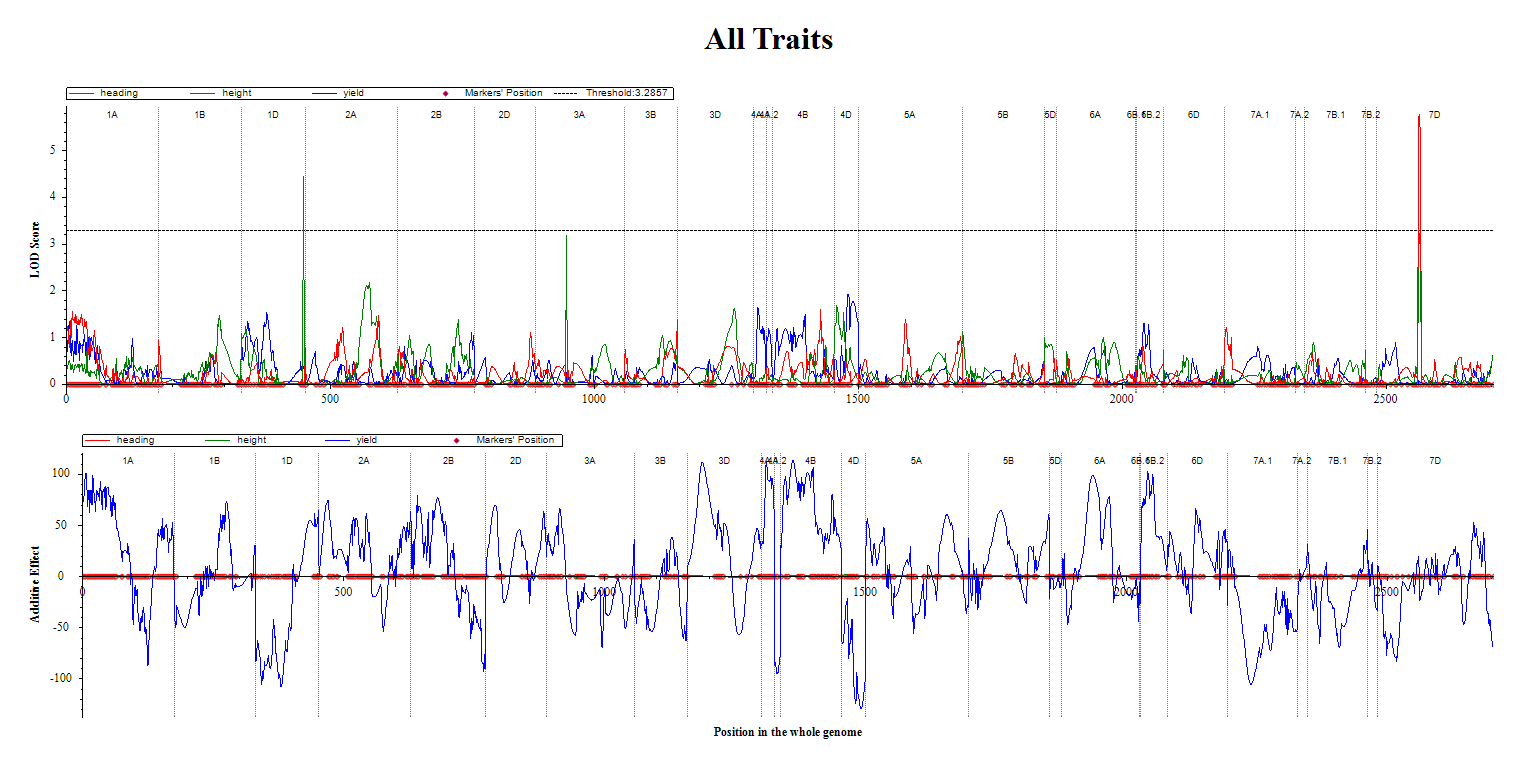


**5)**

**6)**


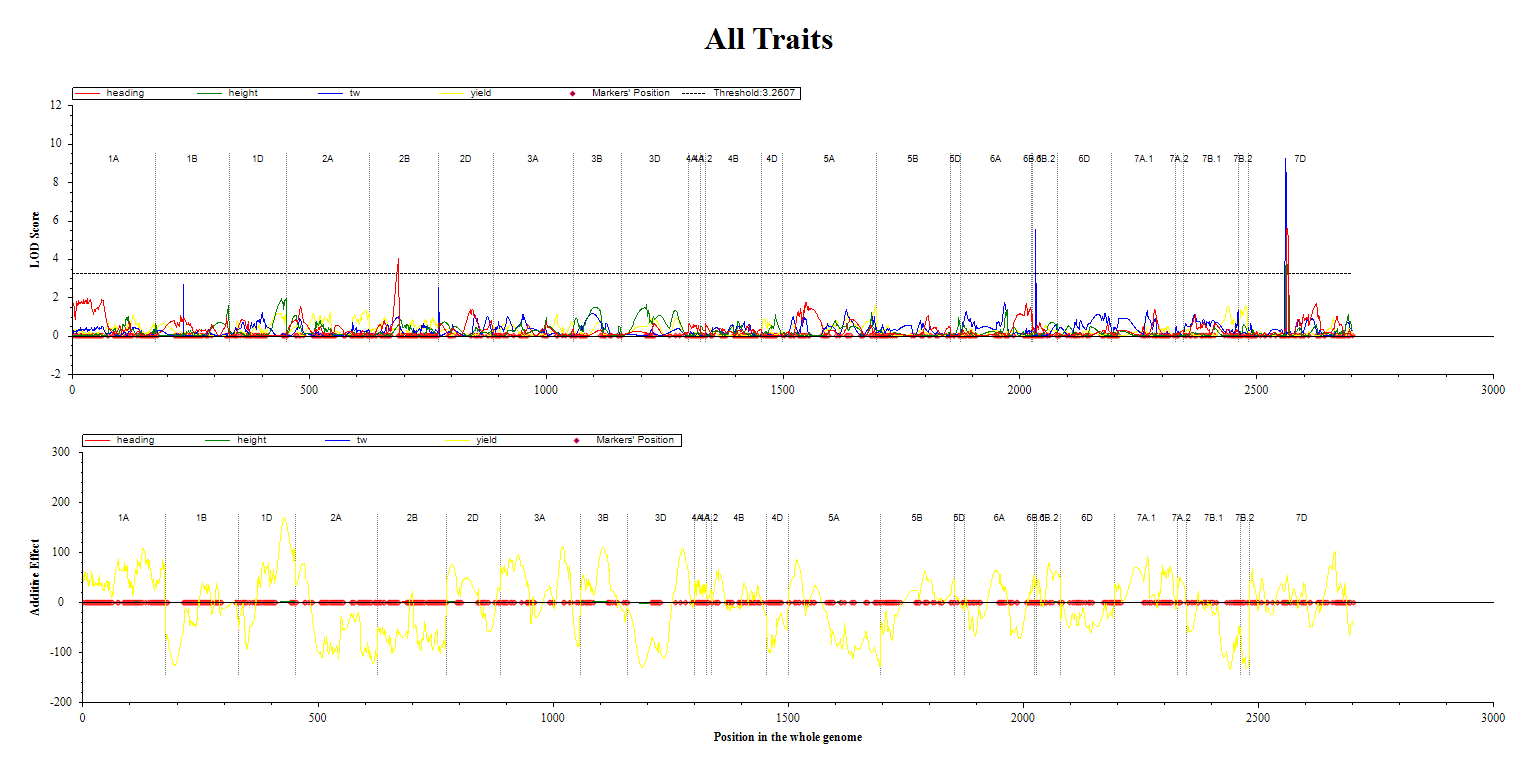


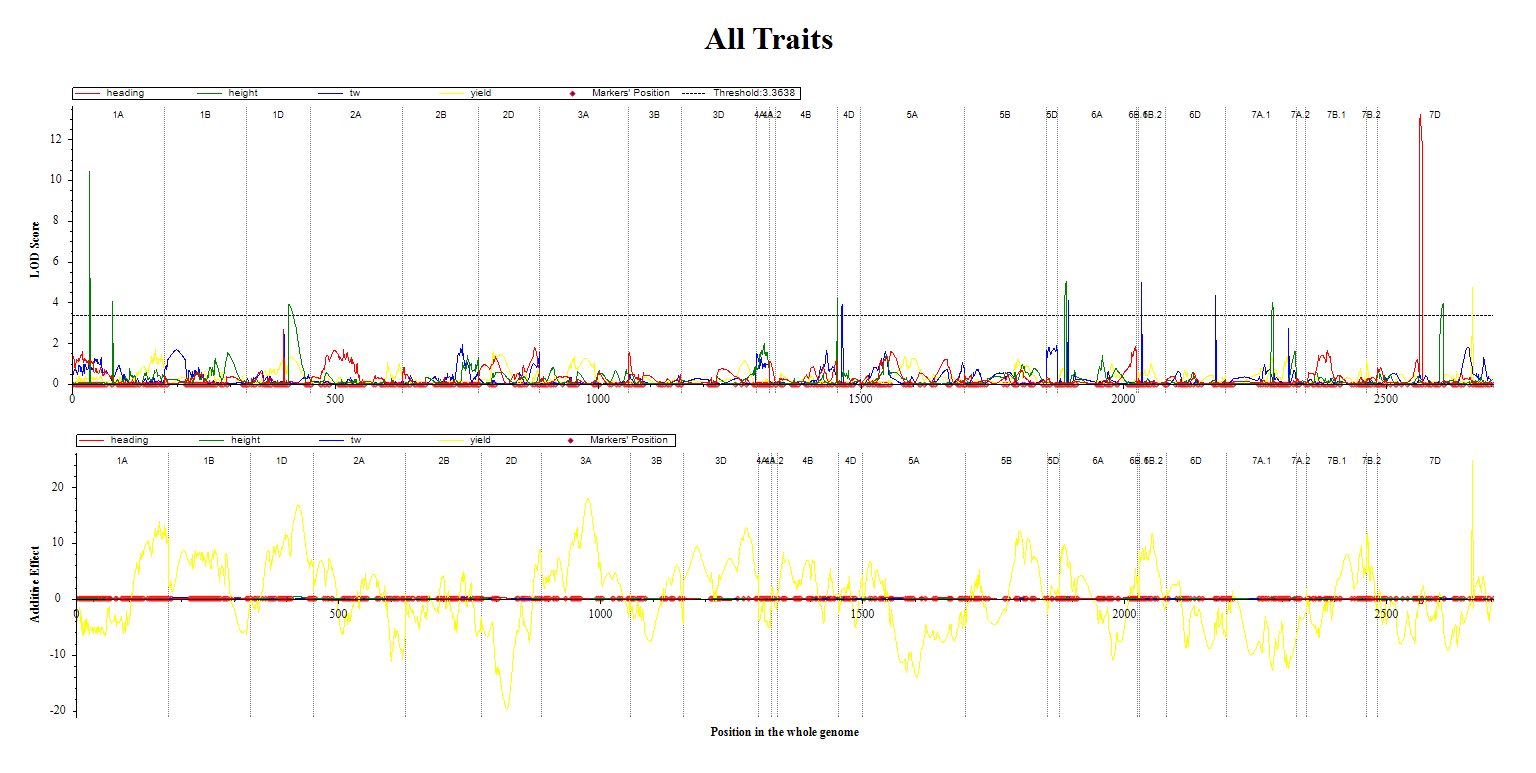


**8)**

**7)**


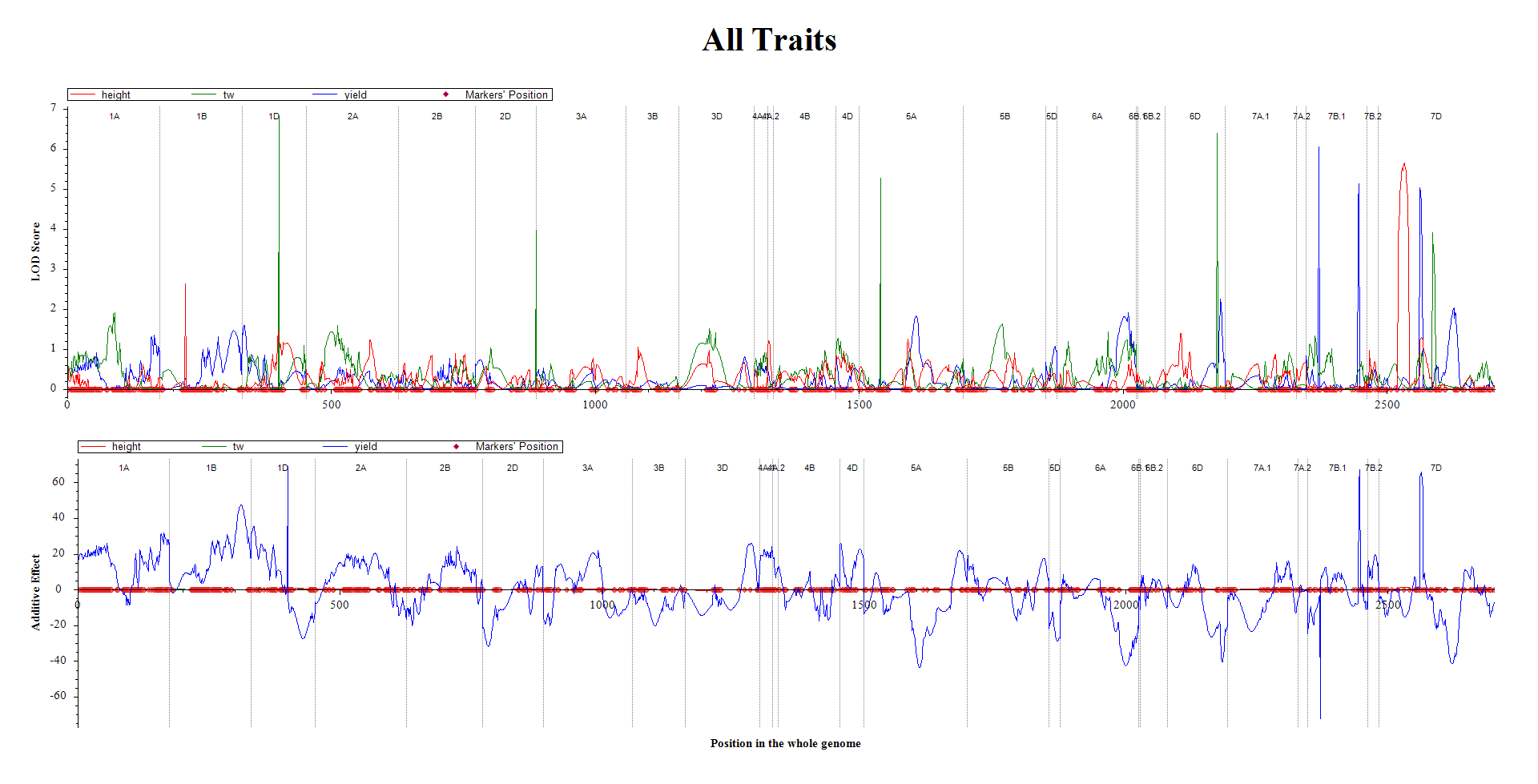


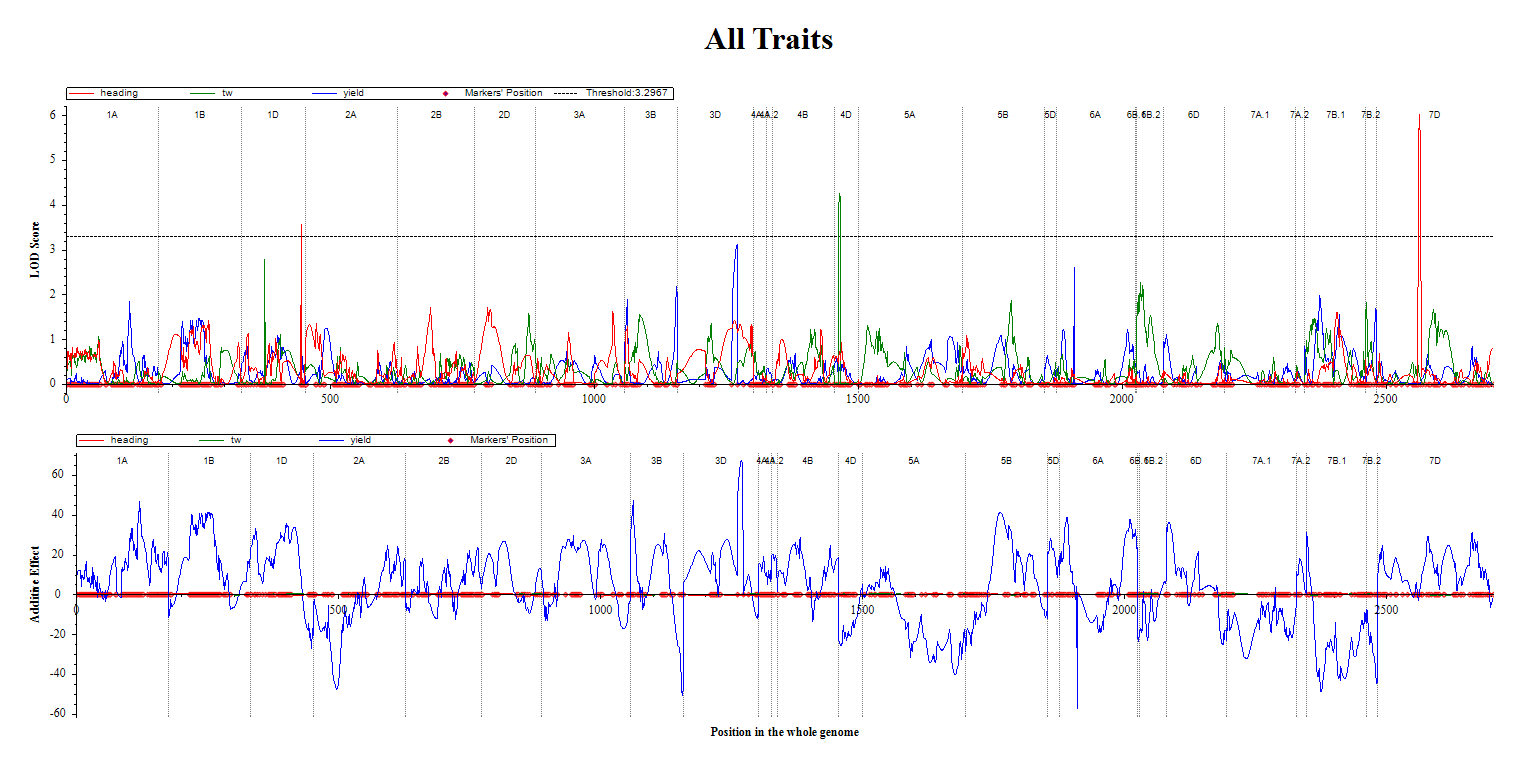


**9)**


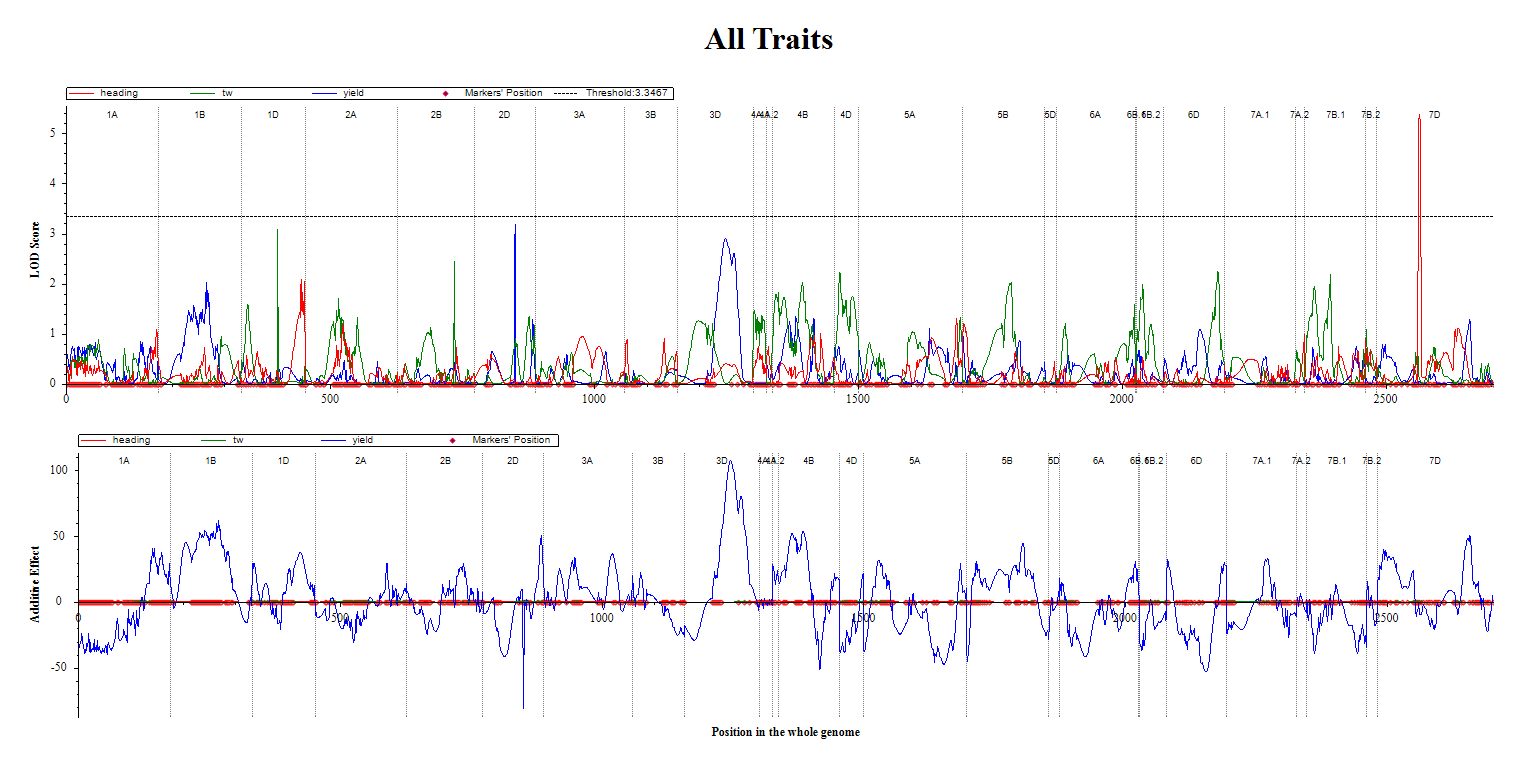


**10)**


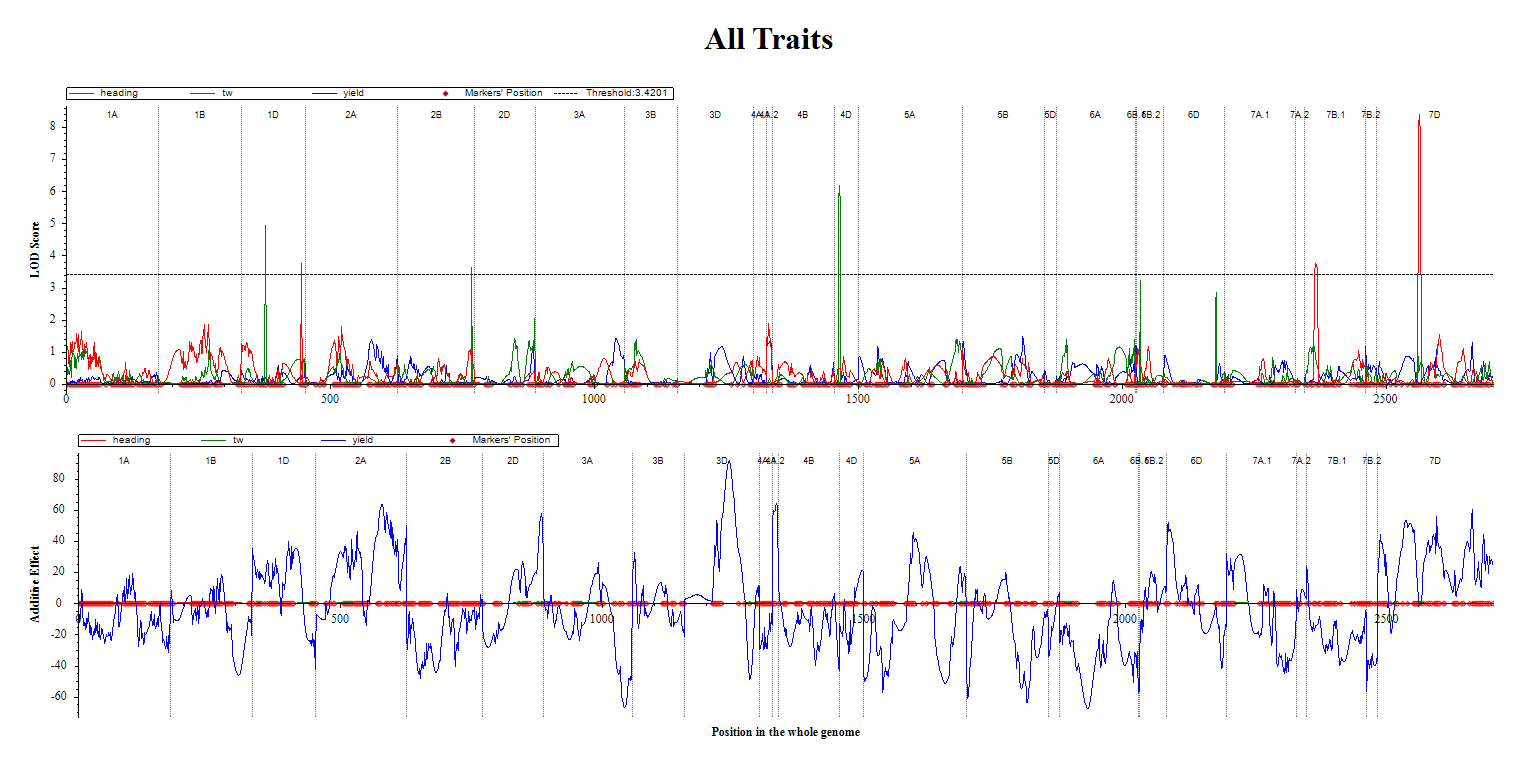


**11)**


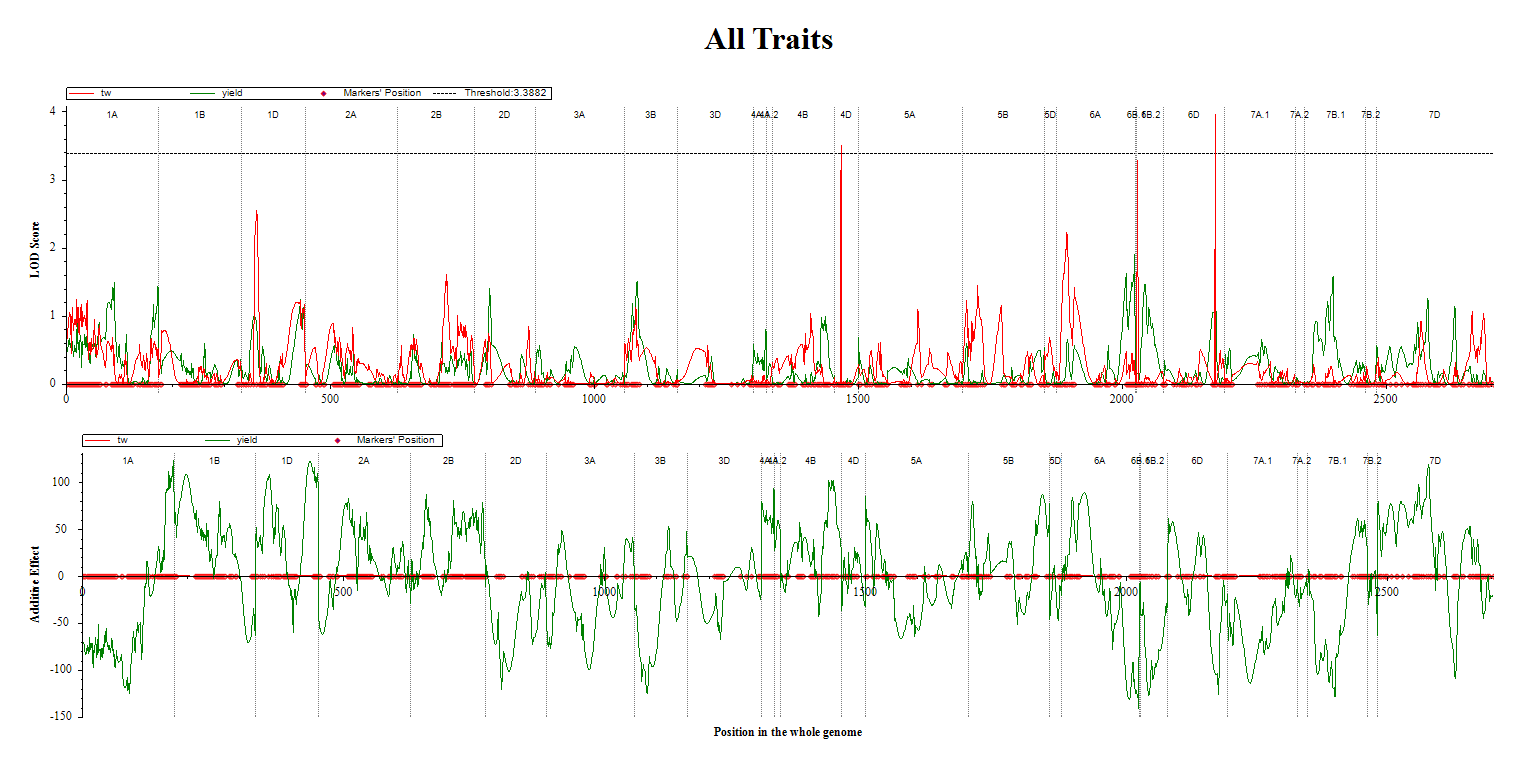


**12)**


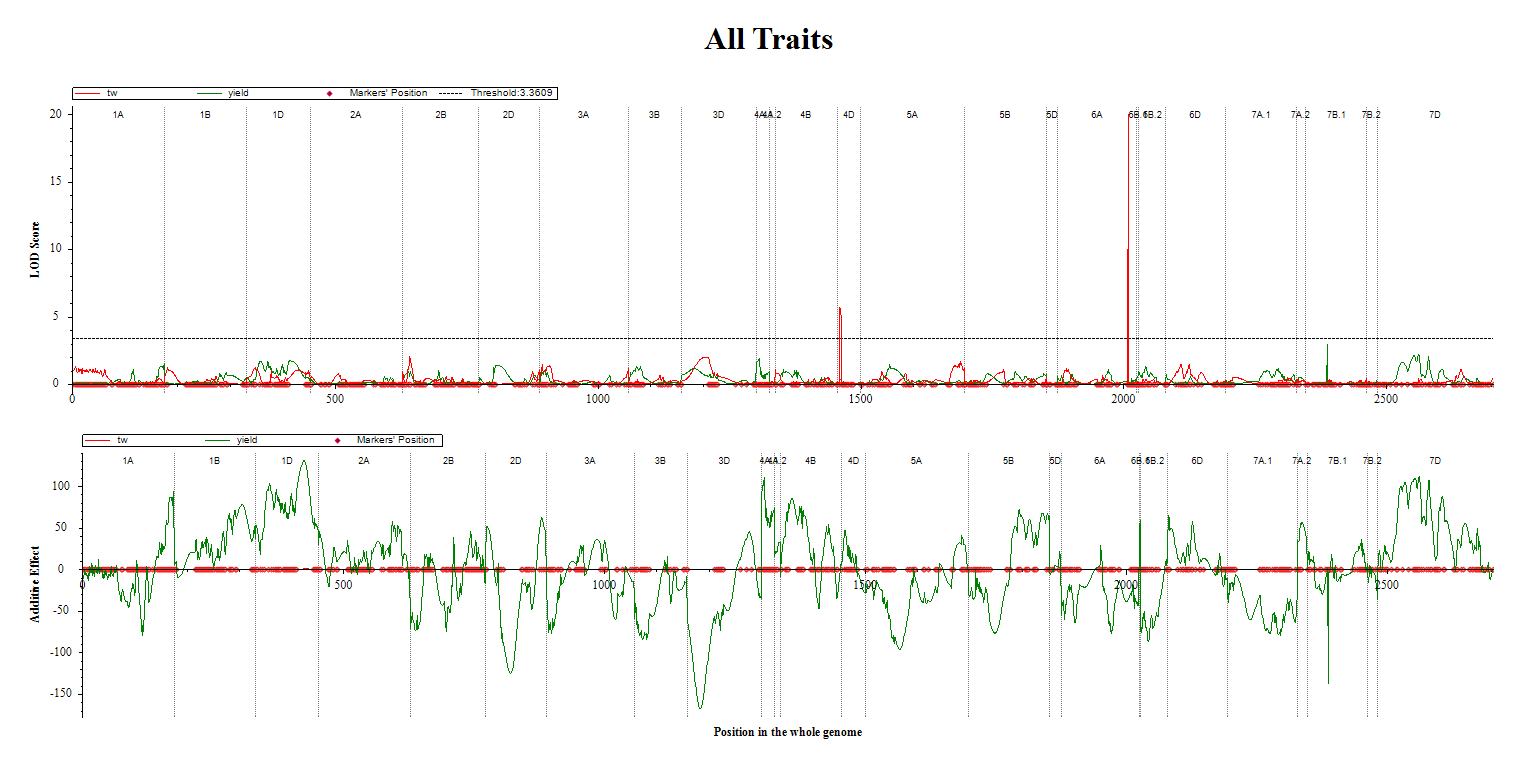


**13)**


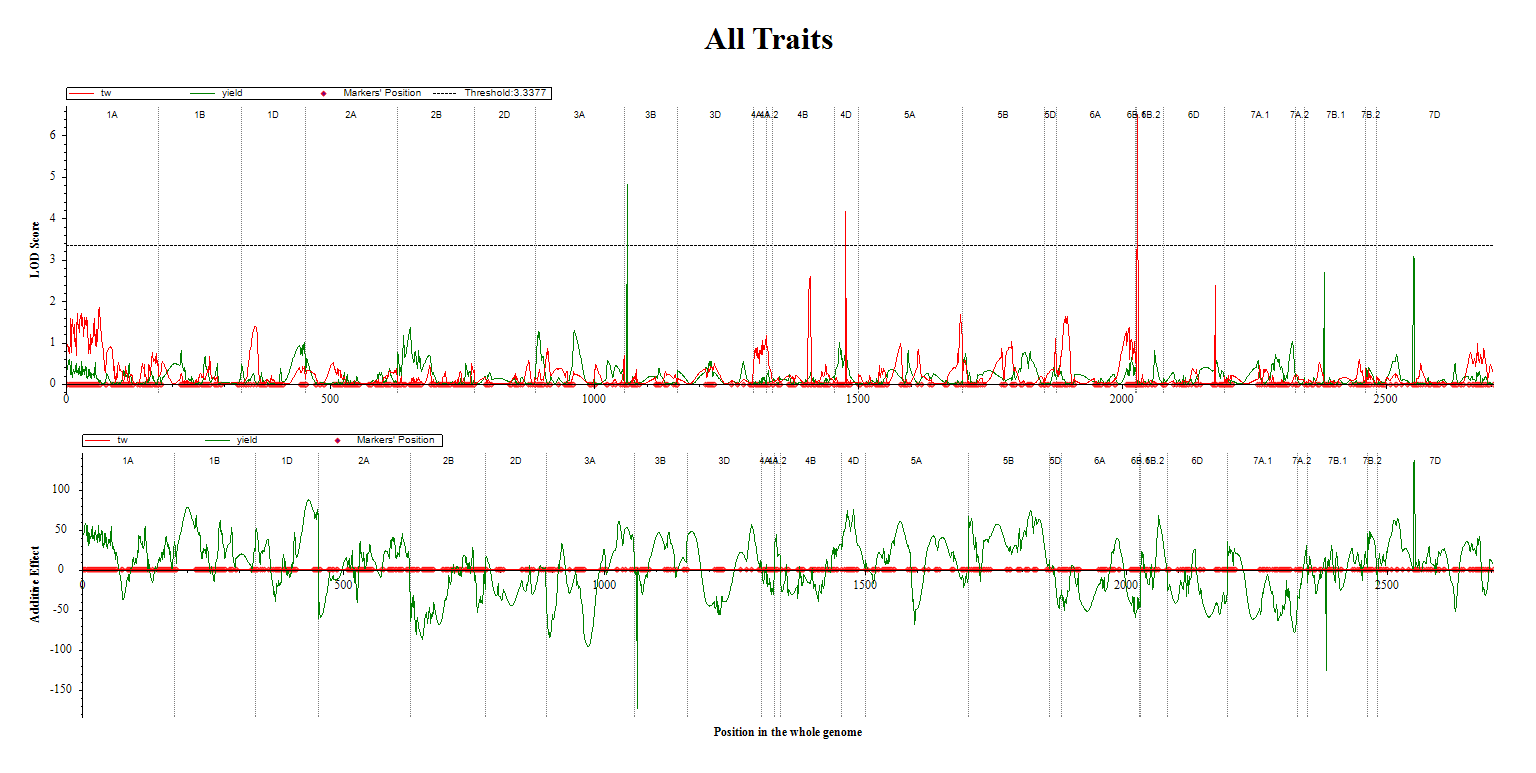


**14)**


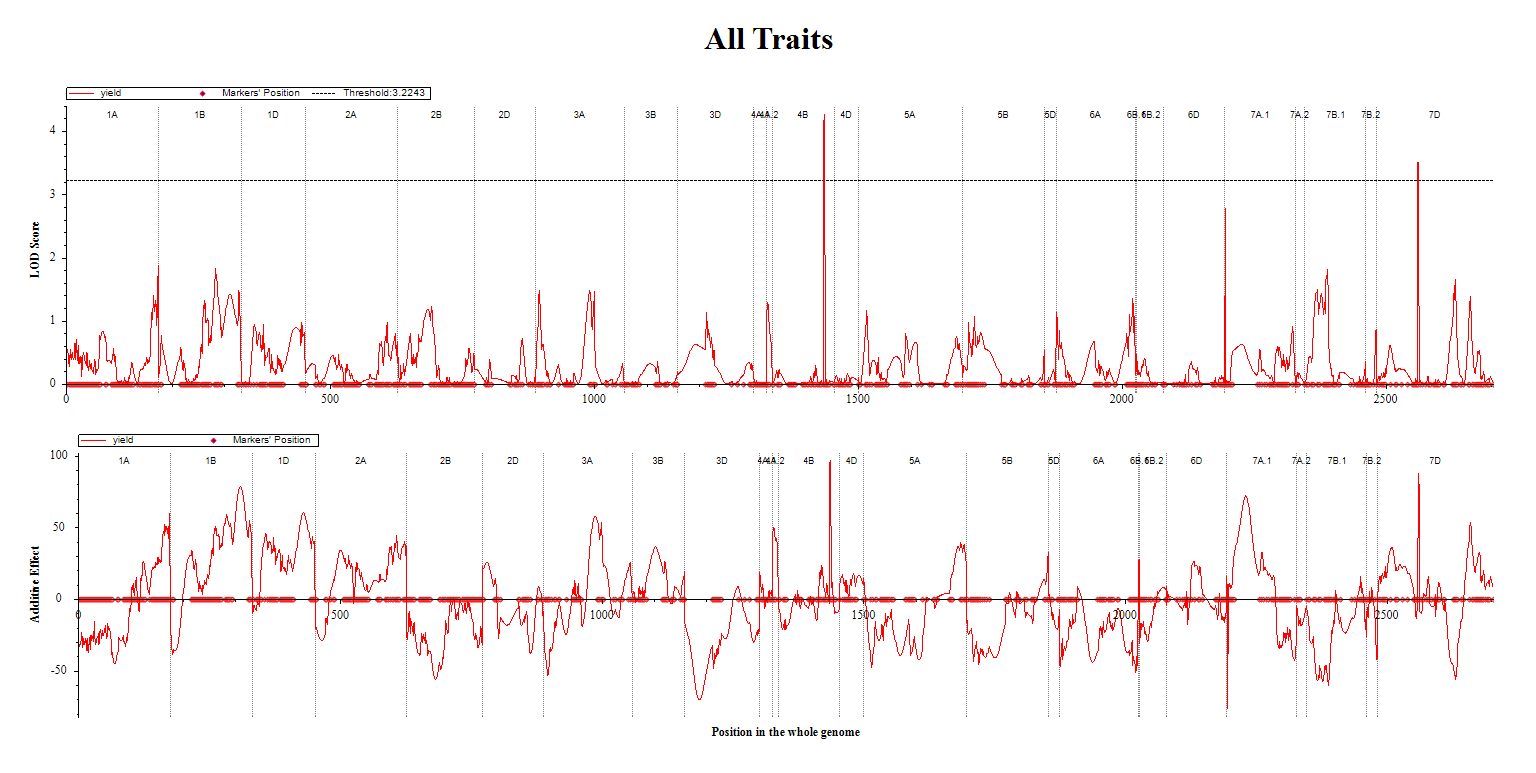


**16)**

**15)**


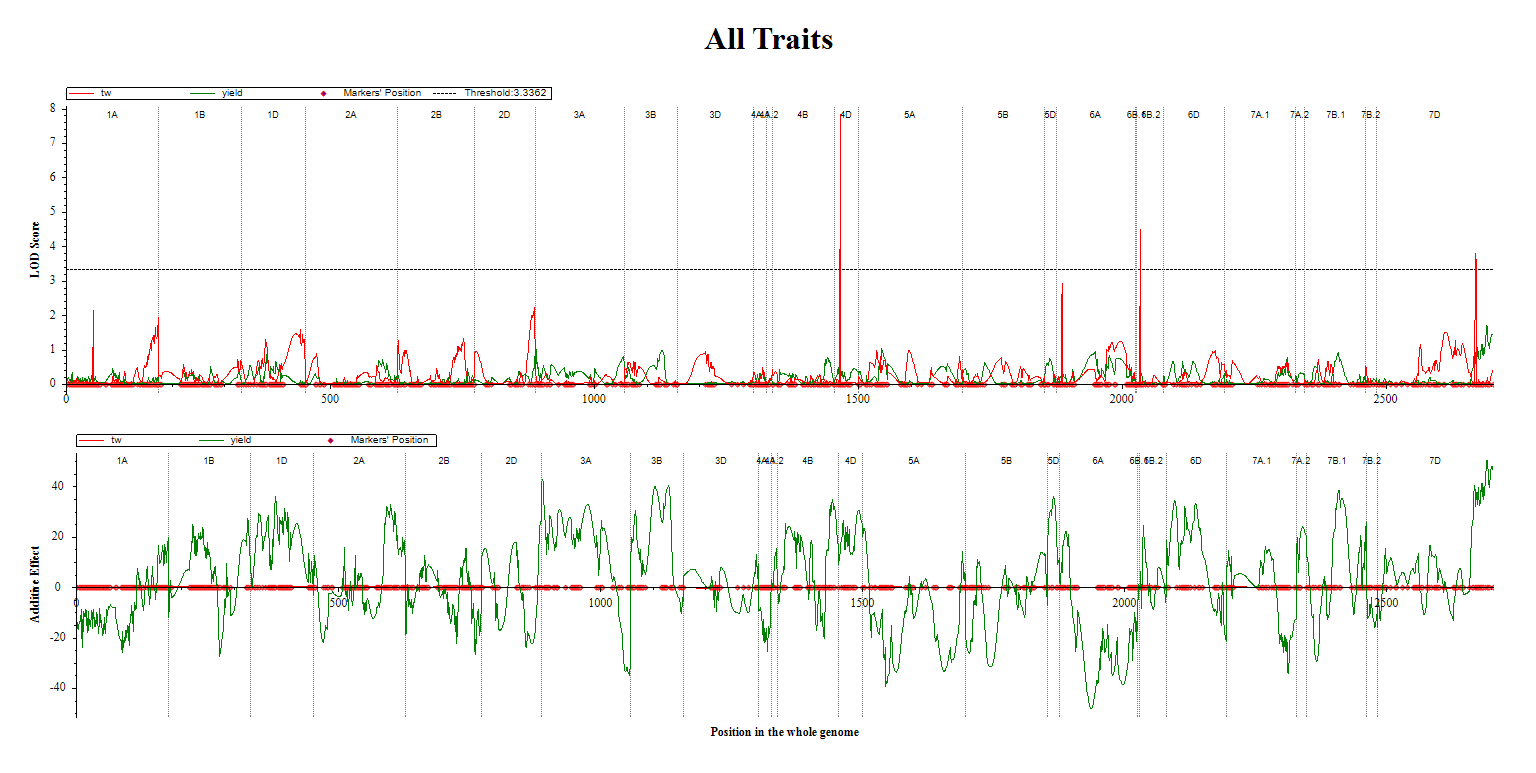


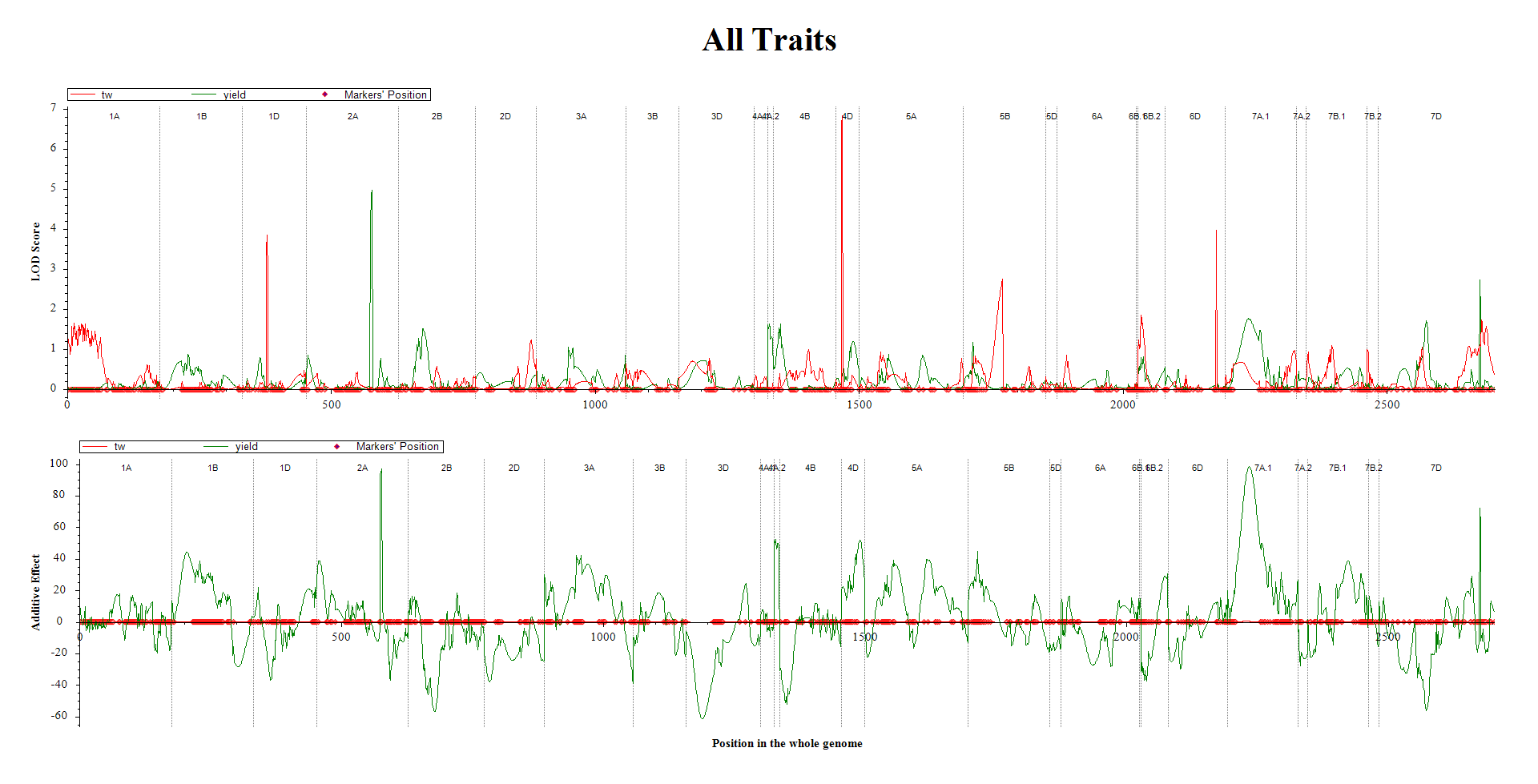


**17)**


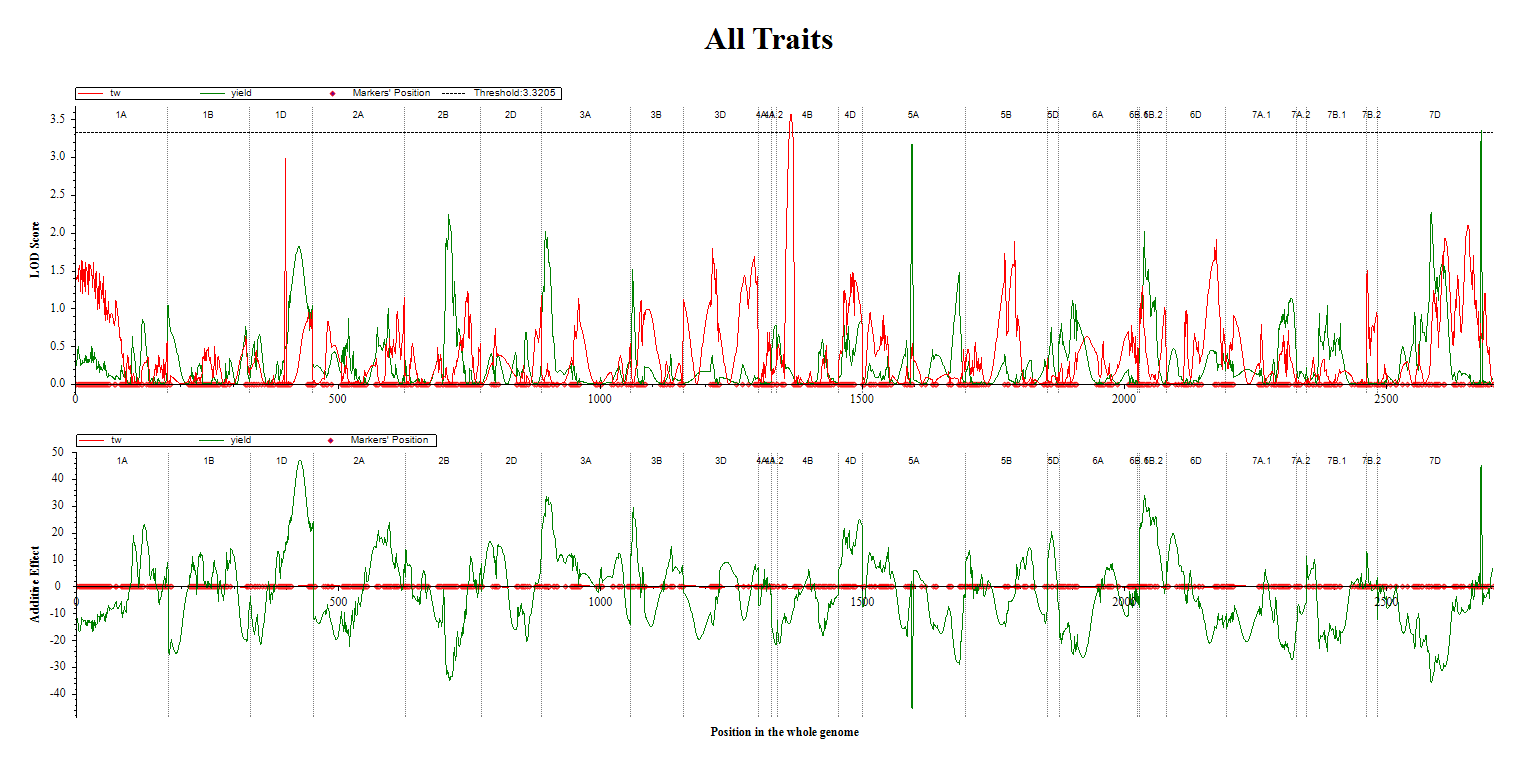


**18)**


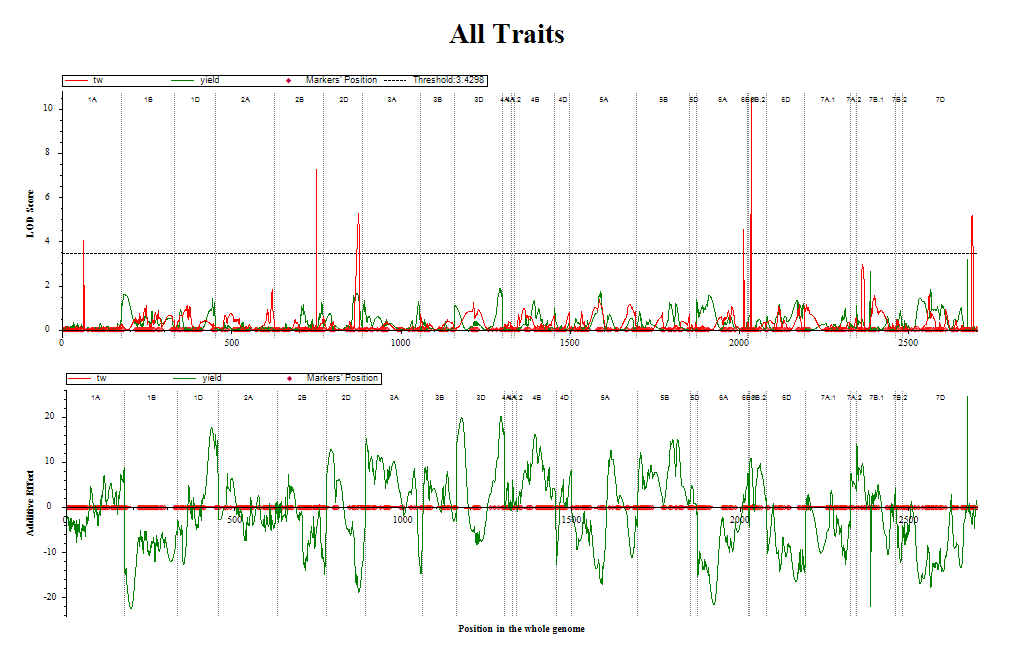


**19)**


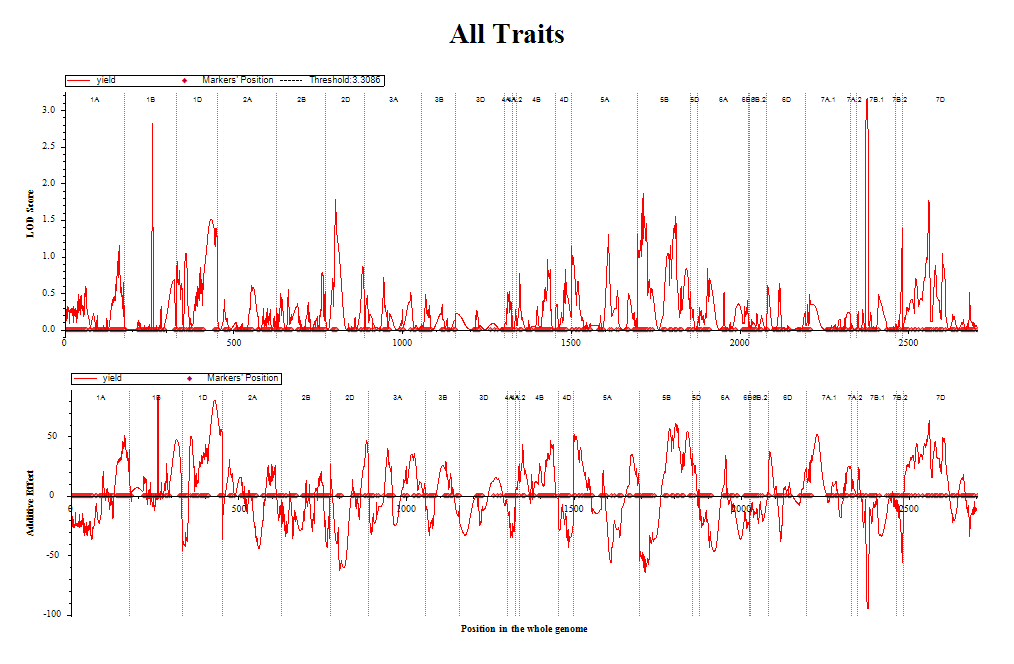


**20)**


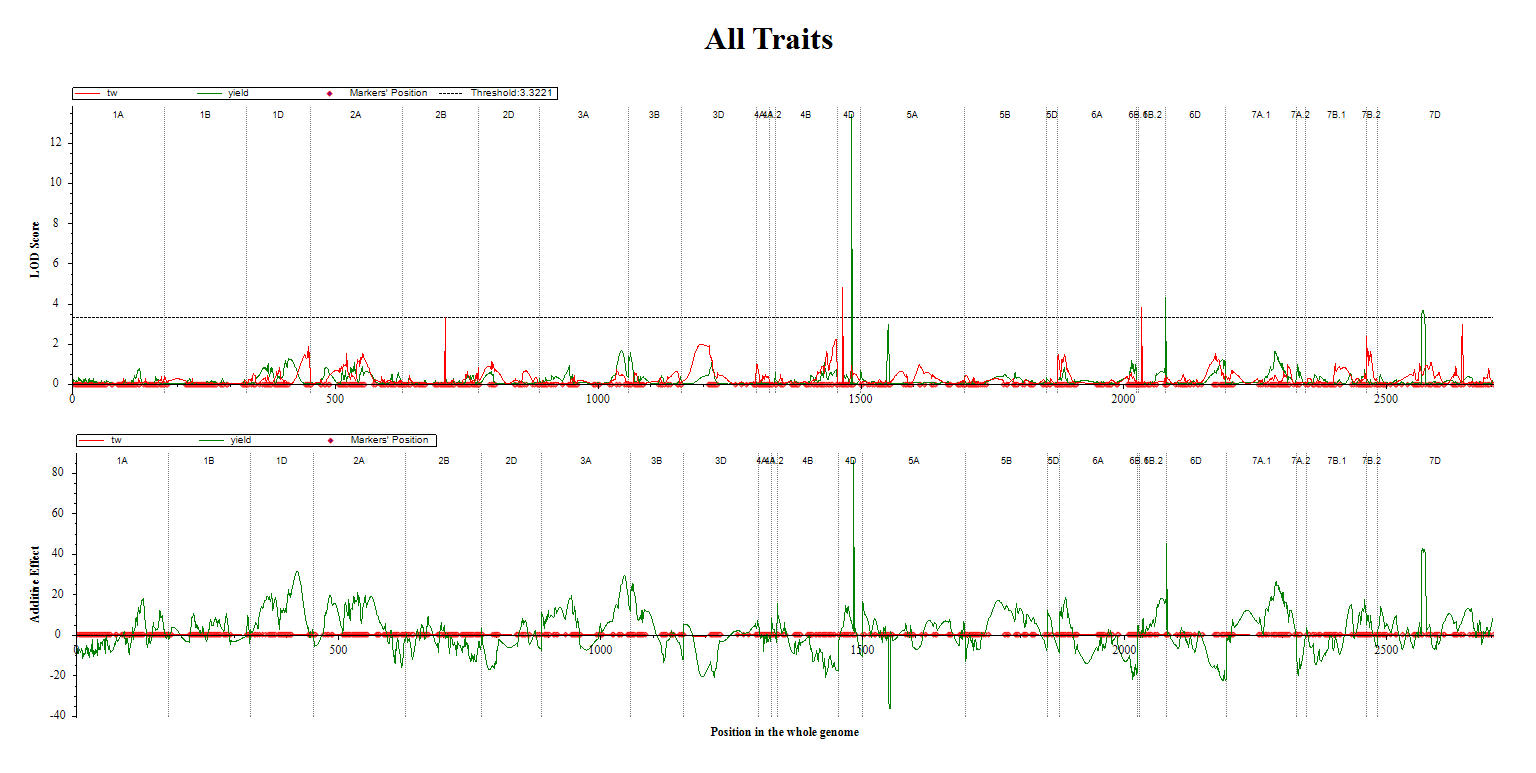


**21)**


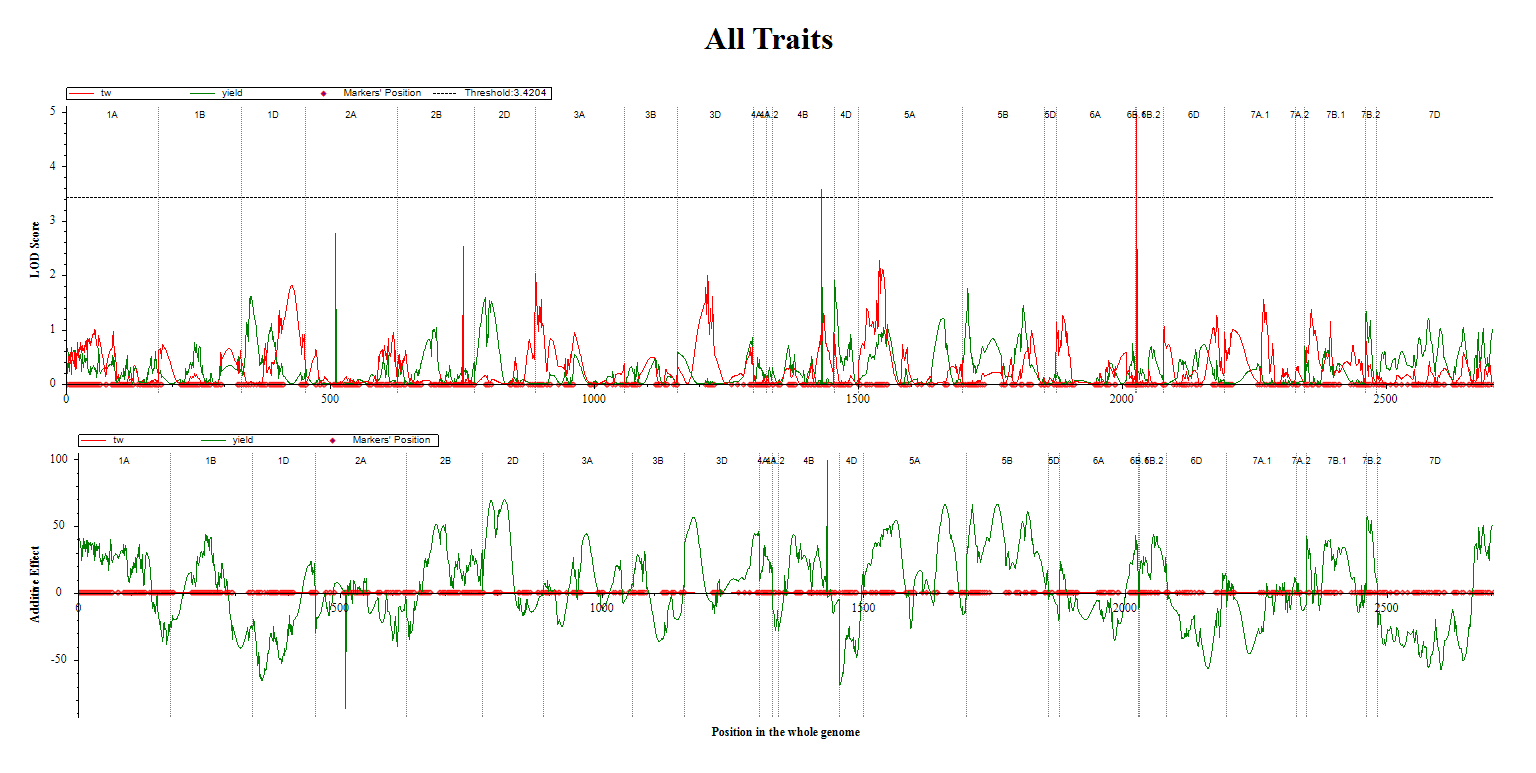


**22)**


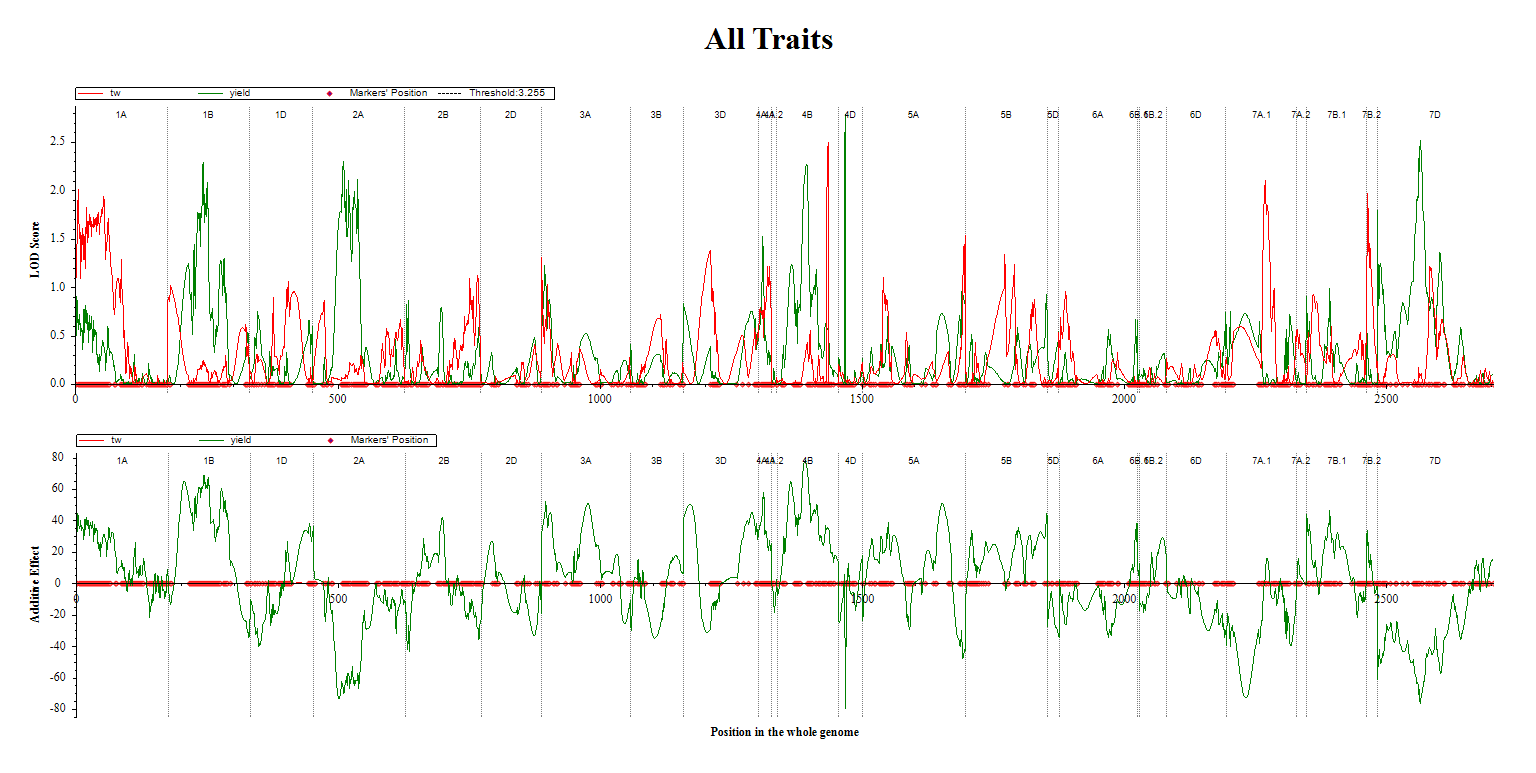


**23)**


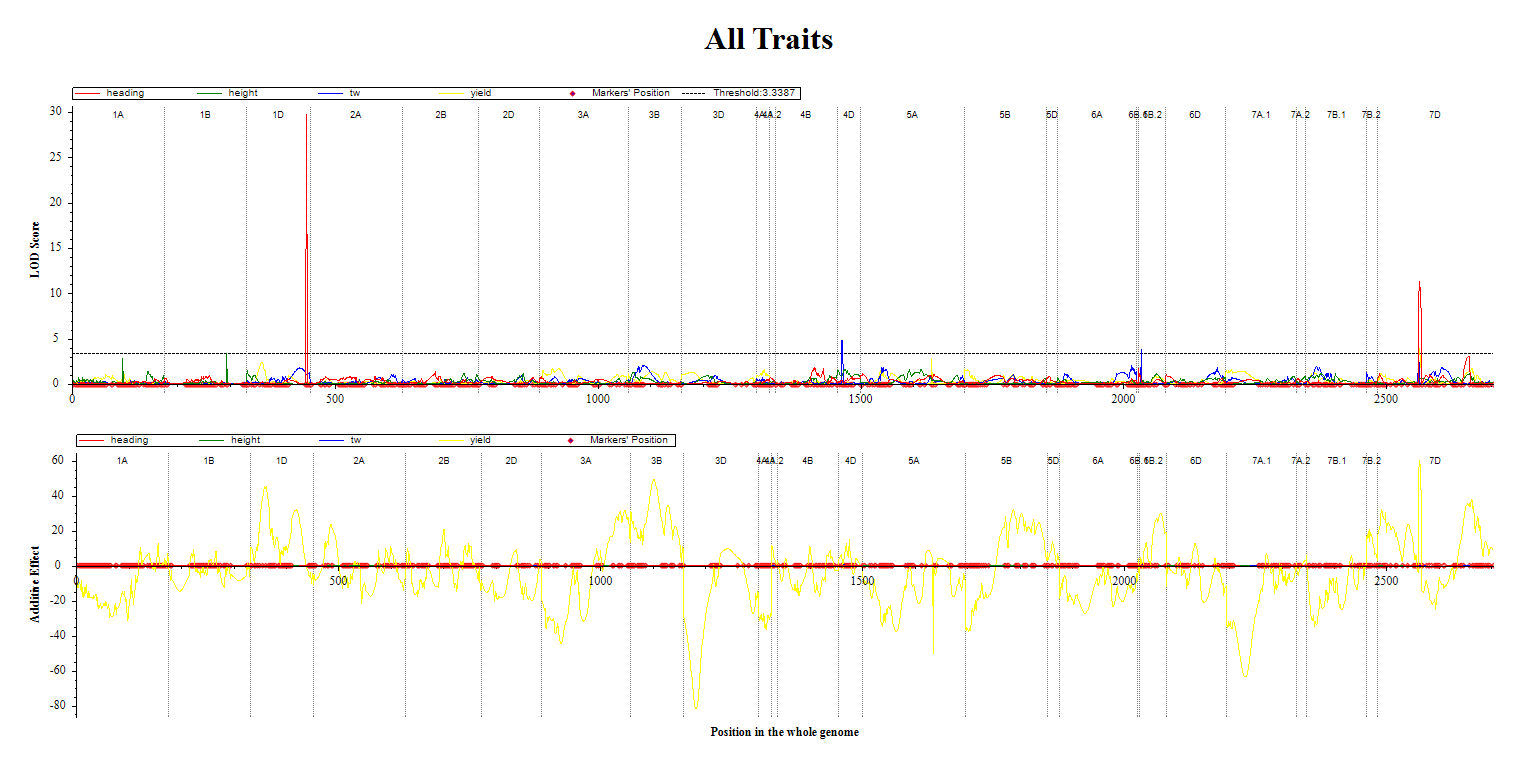


**24)**


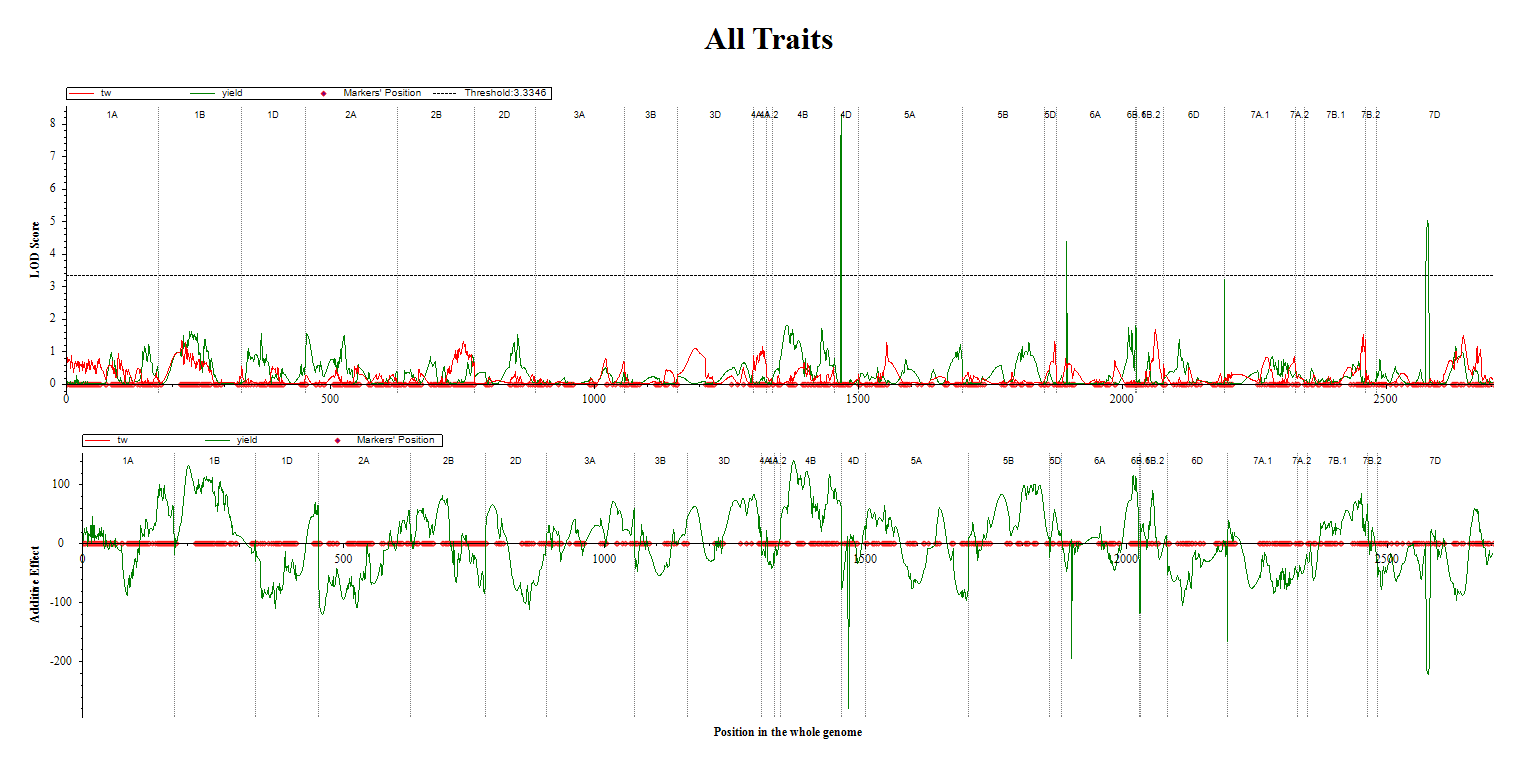


**25)**


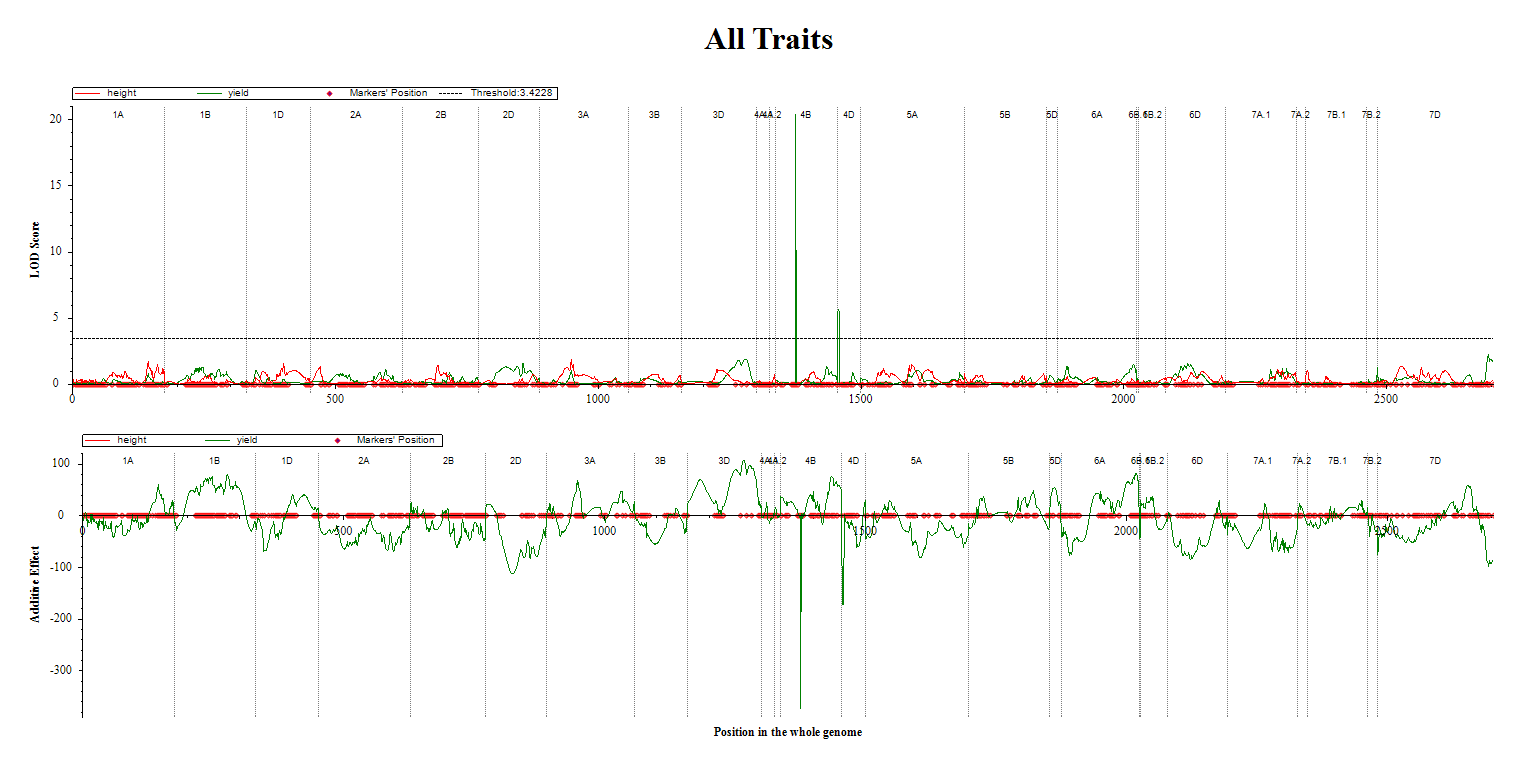


**26)**


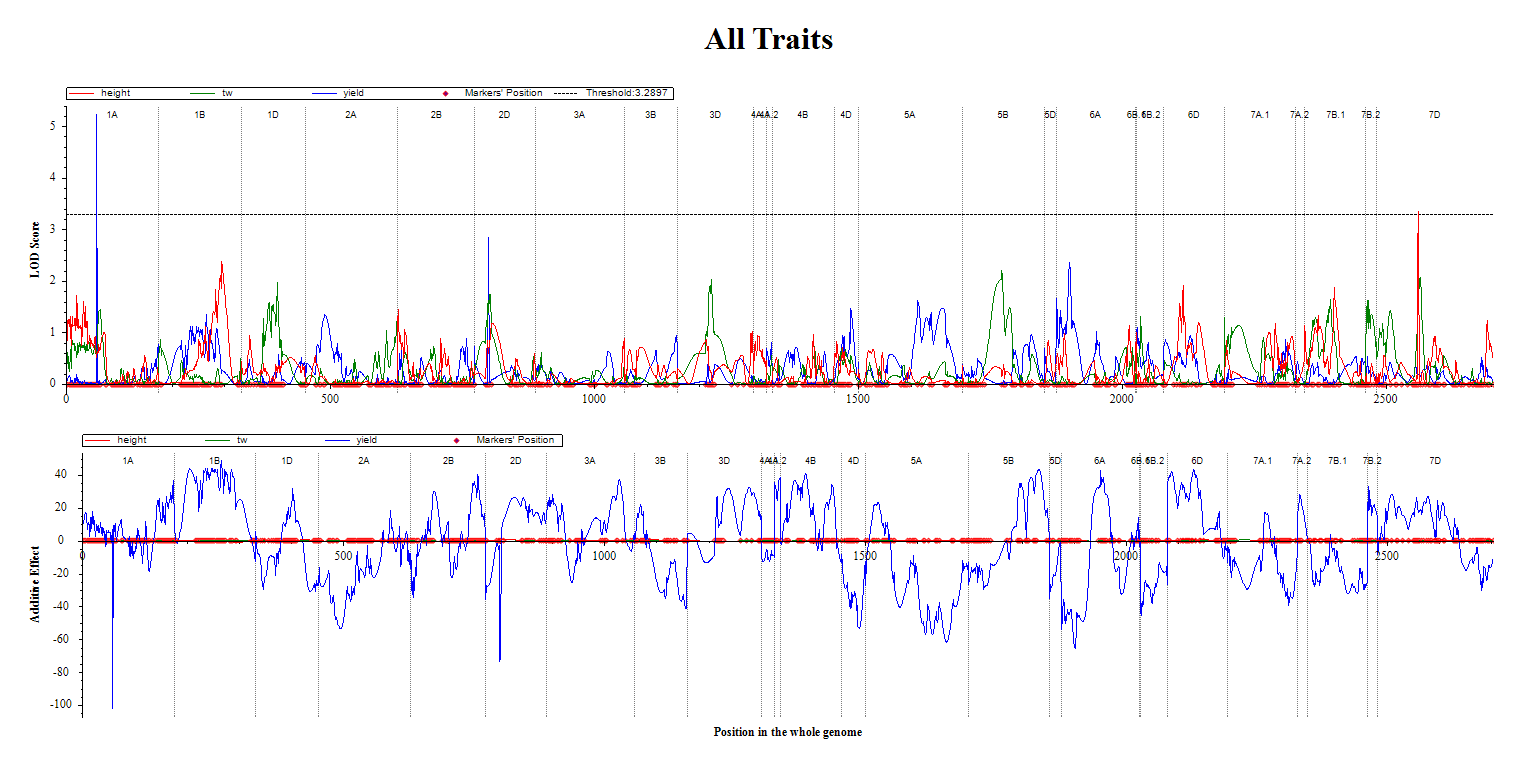


**27)**

Supplemental Fig. S3 LOD profile of additive (LOD(A)), additive-by-environment (LOD(AbyE)), and sum of additive and additive-by-environment (LOD) QTLs detected in the multi-environment QTL analysis for a) heading days, b) plant height, c) test weight, d) grain yield. LOD profile with chromosomal position is shown on the x-axis (cM of 25 LGs) and LOD score and additive effects for traits with significant QTL on the y-axis.


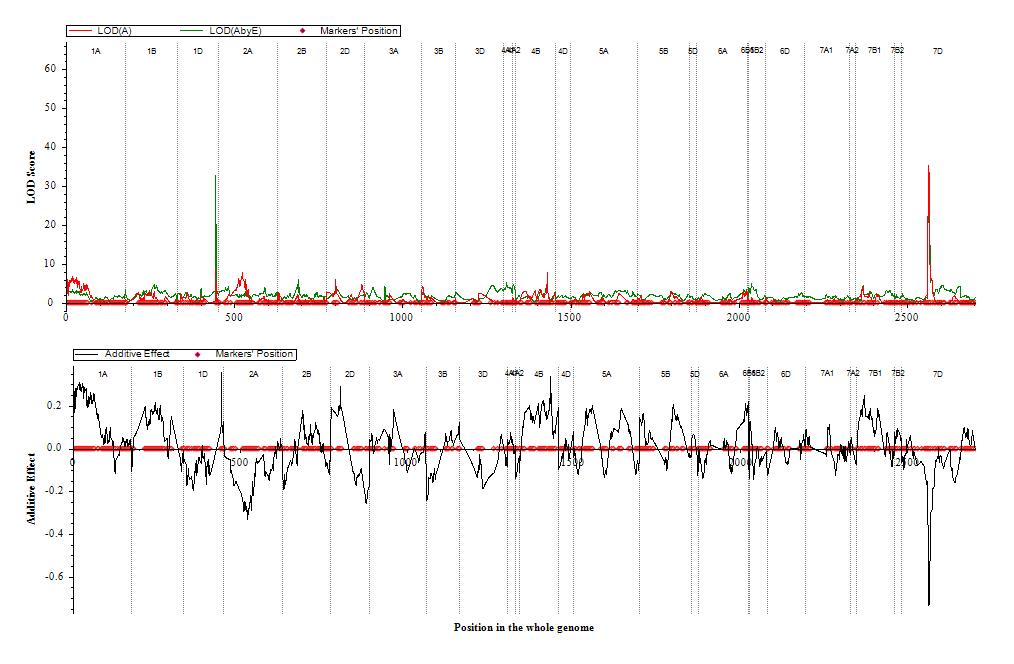


**a)**


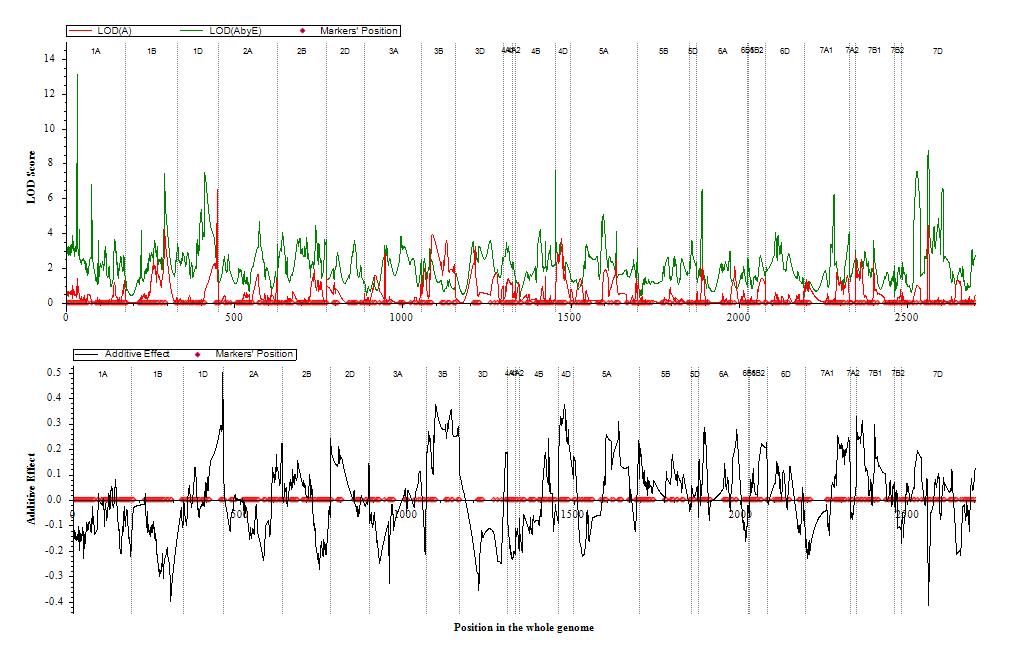


**b)**


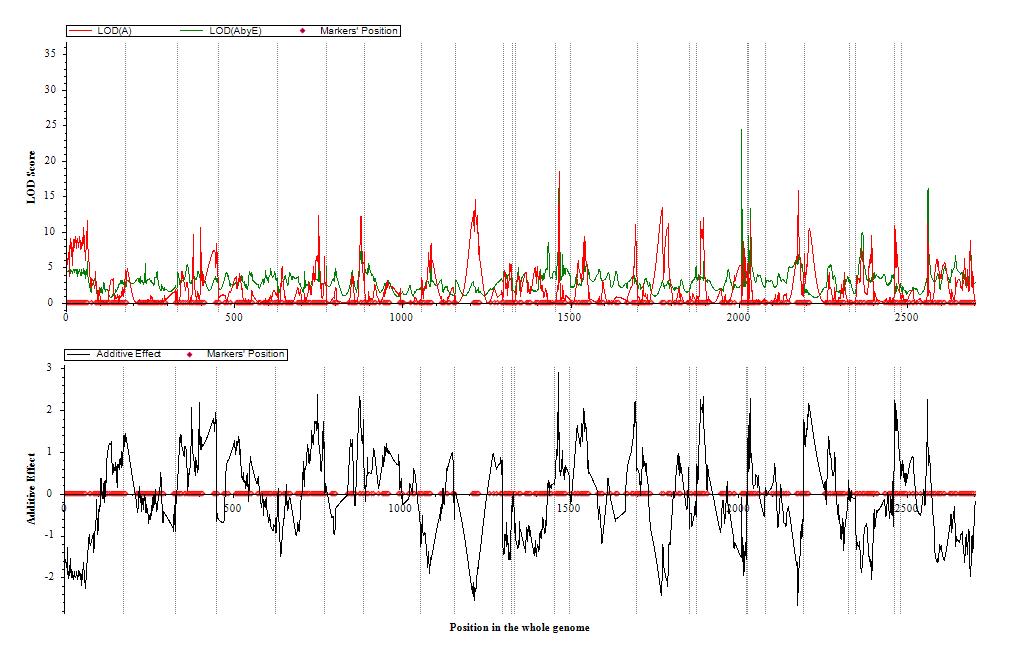


**c)**


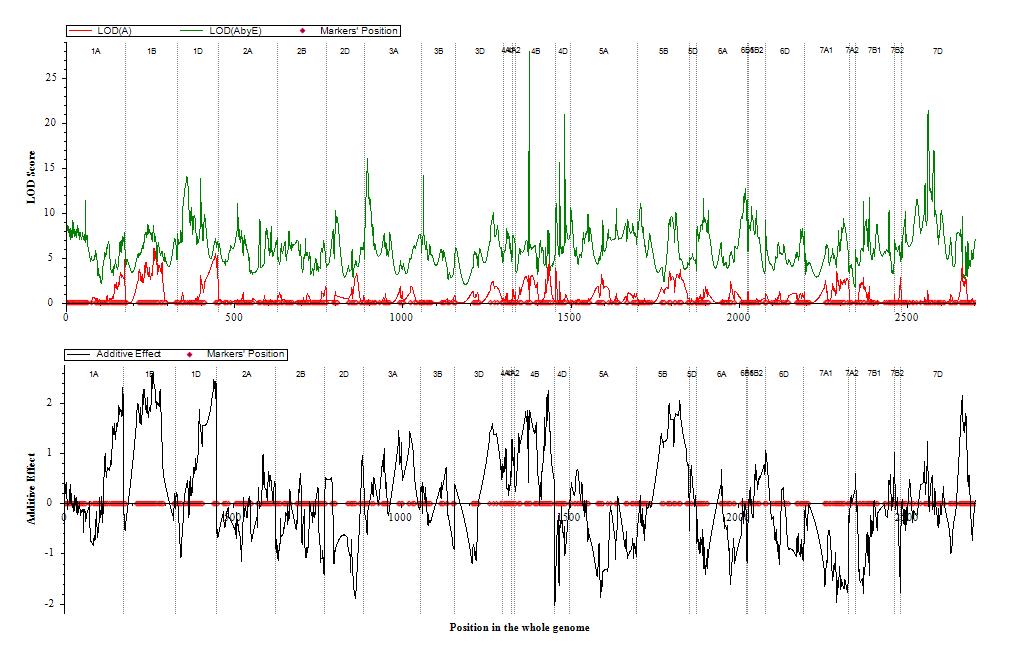


**d)**


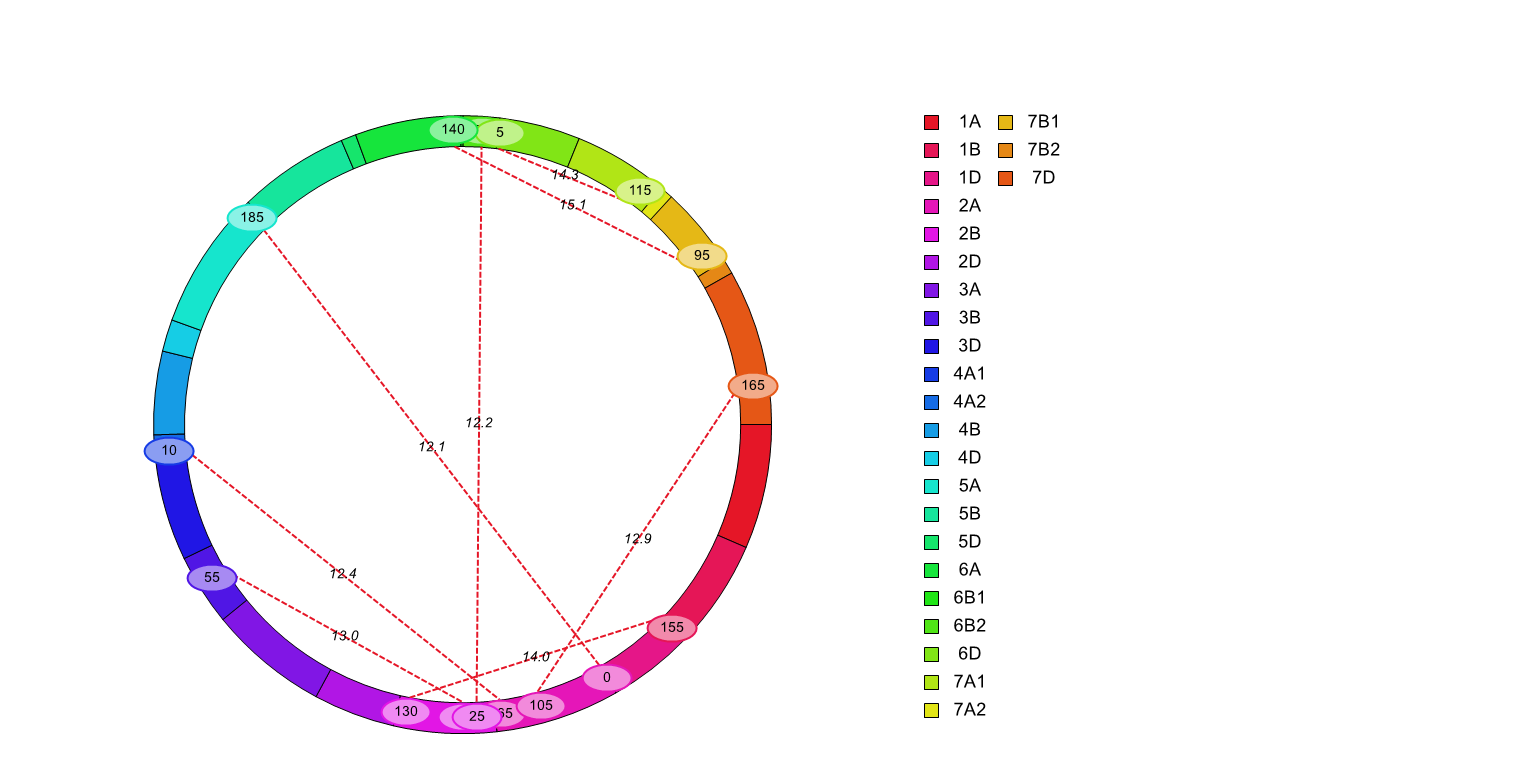
Supplemental Figure S4 Epistatic interaction between QTLs for four traits. a) heading date, b) plant height, c) test weight, d) grain yield. LOD thresholds were 12 for HT and HD and those for test weight and yield were 15. as determined by permutation. QTL interaction LOD values are shown along the dashed line connecting two linkage groups. Linkage groups are color coded as shown on the right side of the figure. The actual bp and linked SNPs are listed in Table S4.


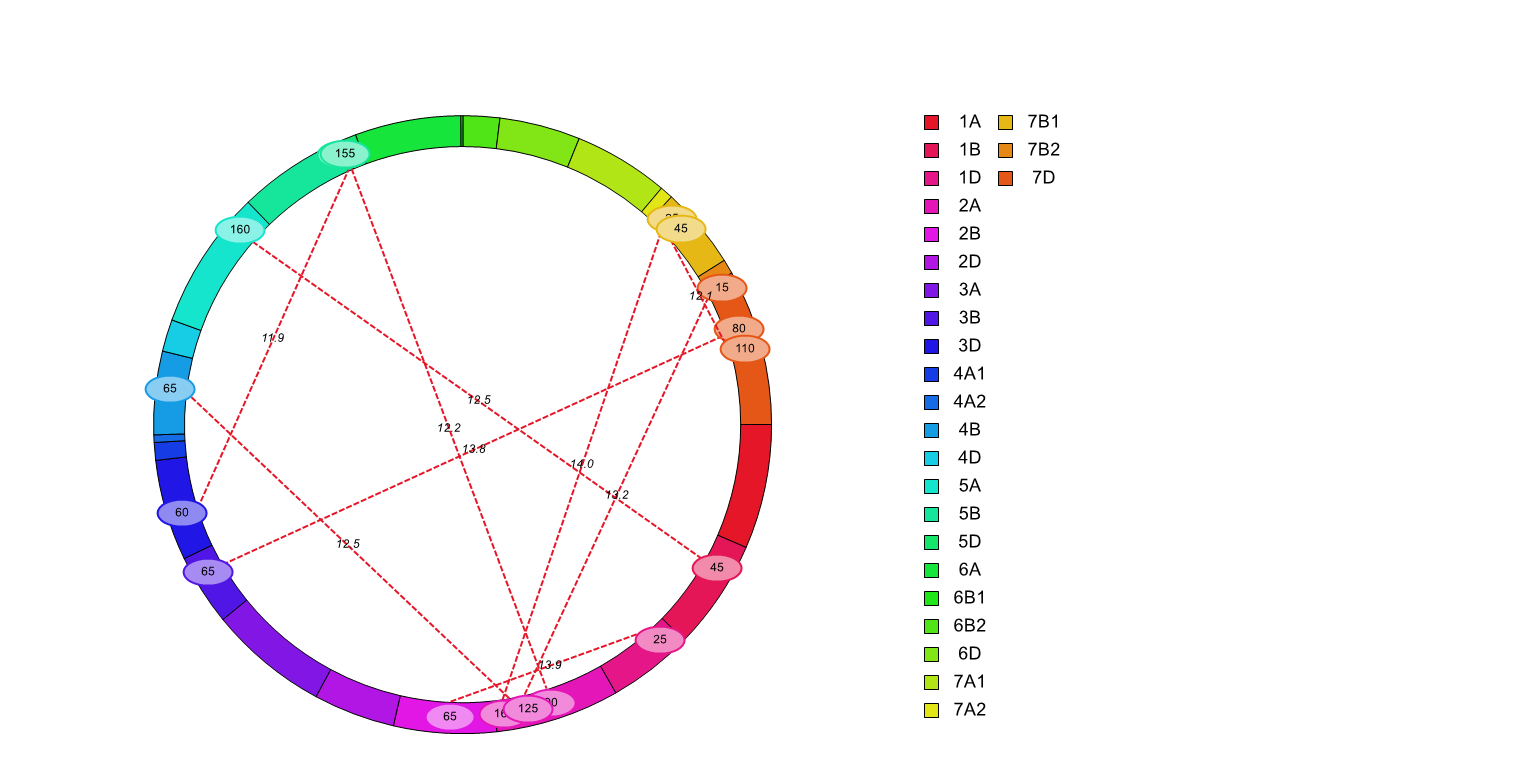


**b)**

**a)**


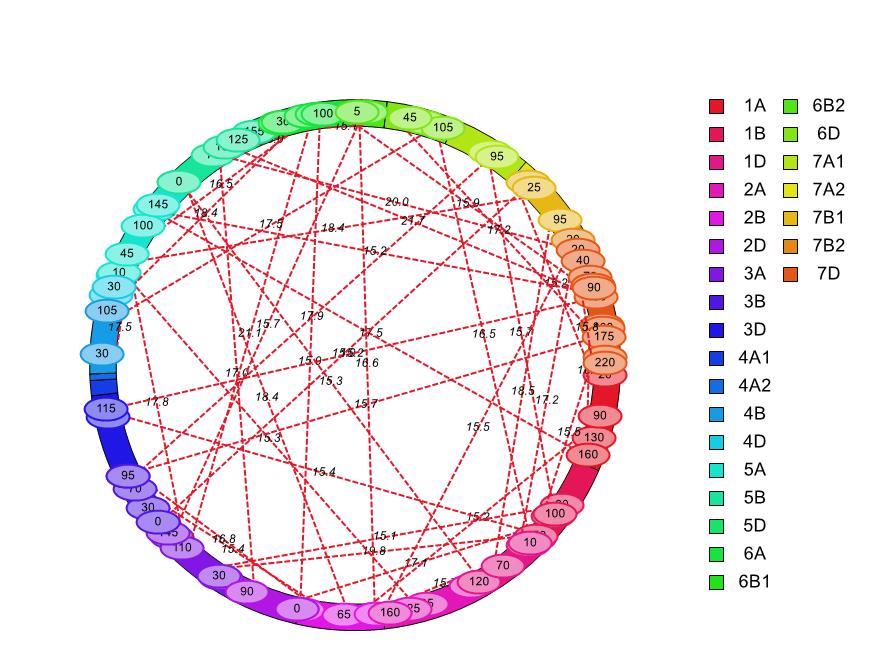

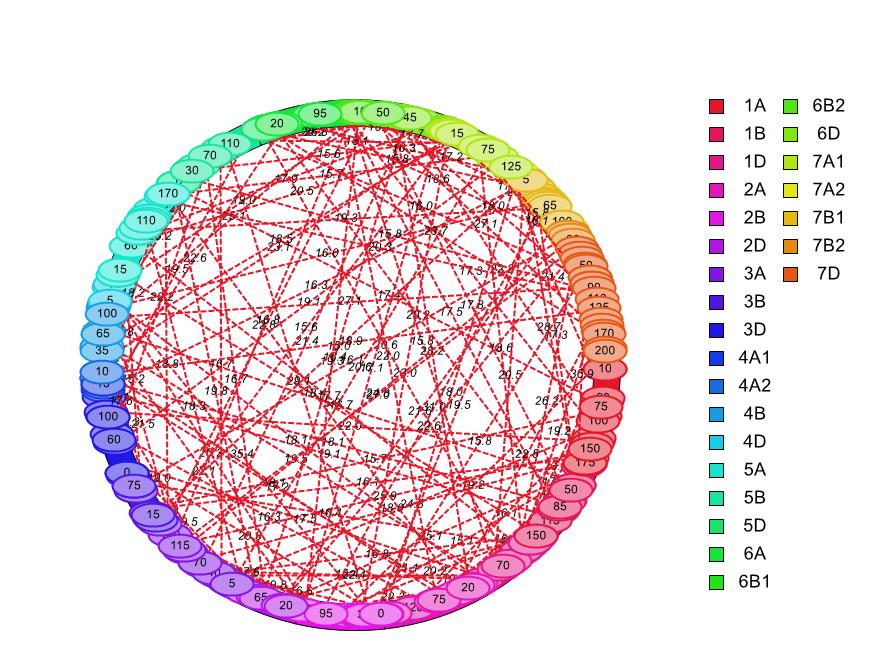


**d)**

**c)**
